# *MISF2* Encodes an Essential Mitochondrial Splicing Cofactor Required for *nad2* mRNA Processing and Embryo Development in *Arabidopsis thaliana*

**DOI:** 10.3390/ijms23052670

**Published:** 2022-02-28

**Authors:** Tan-Trung Nguyen, Corinne Best, Sofia Shevtsov, Michal Zmudjak, Martine Quadrado, Ron Mizrahi, Hagit Zer, Hakim Mireau, Oren Ostersetzer-Biran

**Affiliations:** 1 Institut Jean-Pierre Bourgin (IJPB), INRAE, AgroParisTech, Paris-Saclay University, 78000 Versailles, France; nguyentantrung_cs@yahoo.com (T.-T.N.); martine.quadrado@inrae.fr (M.Q.); 2 Department of Plant and Environmental Sciences, The Alexander Silberman Institute of Life Sciences, The Hebrew University of Jerusalem, Givat-Ram, Jerusalem 91904, Israel; cb1139@waksman.rutgers.edu (C.B.); sofia.shevtsov@gmail.com (S.S.); michalzm@gmail.com (M.Z.); ron.mizrahi1@mail.huji.ac.il (R.M.); hagit.zer@mail.huji.ac.il (H.Z.)

**Keywords:** group II intron, splicing, PPR, respiration, complex I, mitochondria, embryogenesis, *Arabidopsis*, angiosperms

## Abstract

Mitochondria play key roles in cellular energy metabolism in eukaryotes. Mitochondria of most organisms contain their own genome and specific transcription and translation machineries. The expression of angiosperm mtDNA involves extensive RNA-processing steps, such as RNA trimming, editing, and the splicing of numerous group II-type introns. Pentatricopeptide repeat (PPR) proteins are key players in plant organelle gene expression and RNA metabolism. In the present analysis, we reveal the function of the *MITOCHONDRIAL SPLICING FACTOR* 2 gene (*MISF2*, *AT3G22670*) and show that it encodes a mitochondria-localized PPR protein that is crucial for early embryo development in *Arabidopsis*. Molecular characterization of embryo-rescued *misf2* plantlets indicates that the splicing of *nad2* intron 1, and thus respiratory complex I biogenesis, are strongly compromised. Moreover, the molecular function seems conserved between MISF2 protein in *Arabidopsis* and its orthologous gene (*EMP10*) in maize, suggesting that the ancestor of MISF2/EMP10 was recruited to function in *nad2* processing before the monocot–dicot divergence ~200 million years ago. These data provide new insights into the function of nuclear-encoded factors in mitochondrial gene expression and respiratory chain biogenesis during plant embryo development.

## 1. Introduction

Mitochondria are key sites of cellular energy metabolism (i.e., ATP production), as well as of the biosynthesis of various essential metabolites. Most modern mitochondria contain vestigial genomes (mtDNA and mitogenome) derived from that of their ancestral bacterial progenitor, which vary quite widely in size between organisms. In plants, angiosperm mtDNAs are remarkably large and complex in structure [1], encoding rRNAs, tRNAs, ribosomal proteins, as well as various subunits of respiratory complexes (CI to CIV), the ATP synthase enzyme (CV), cofactors of the cytochrome c biogenesis (CCM) machinery, and at least one component of the twin-arginine protein translocation system [2].

In *Arabidopsis*, the oxidative phosphorylation (OXHPOS) machinery is composed of >100 different subunits, most of which are encoded by nuclear loci and about 20 of which are expressed from the mitogenome. Complex I (CI, or NADH-ubiquinone oxidoreductase), which catalyzes NADH dehydrogenation and electron transfer to coenzyme Q10 (CoQ10, or ubiquinone), is the largest and most complicated enzyme of the respiratory chain system [3]. In plants, CI is composed of more than 50 different subunits, 9 of which are encoded in the mitochondria (i.e., Nad1, Nad2, Nad3, Nad4, Nad4l, Nad5, Nad6, Nad7, and Nad9) [4]. These are assembled into two main sub-domains known as the ‘membrane arm’ and ‘peripheral arm’ of the holo-CI enzyme [5]. Nad1, Nad2, Nad3, Nad4, Nad4L, Nad5, and Nad6 belong to the membrane arm, which functions in proton pumping across the cristae membranes, while Nad7 and Nad9 are incorporated together with other nuclear-encoded subunits into the peripheral arm of CI, which is mainly associated with the transfer of electrons from NADH to ubiquinone. The biogenesis of the respiratory chain machinery involves various mechanisms for regulating the expression of subunits that are encoded by two physically separate genetic compartments [3,6,7,8]. Mutants affected in the expression of mitochondria-encoded CI subunits often show altered growth and developmental phenotypes, some of which contain developmentally arrested embryos [9,10]. Yet, the identity of the factors and pathways involved in these regulations still await further analysis.

The expression of mitochondrial genes in plants involves extensive RNA-processing steps. These include transcript trimming, RNA editing, and the removal of many group II intron sequences that interrupt the coding sequence of several essential genes [9,11,12,13]. These RNA processing steps are essential for mt-RNAs to synthesize the protein they encode. Group II introns are defined by a secondary structure formed by six stem-loop domains (D1 to D6). The excision of canonical group II introns relies on proteins encoded by the introns themselves (i.e., IEPs, or maturases) [14,15], whereas the splicing of group II introns in plant organelles involves a repertoire of nuclear-encoded factors that assist with the splicing reactions and which may serve as key control points in plant mitochondrial gene expression [1,16,17,18]. These belong to diverse families of RNA binding factors. A few are related to maturases [18,19], whereas others are identified as, for e.g., RNA helicases [20,21,22], PORR-related proteins [23], and relevantly to our study, pentatricopeptide repeat (PPR) proteins [24].

The PPR family constitutes a large protein family in land plants, with approximately 450 members identified in *Arabidopsis* and about 490 genes in maize [25,26,27]. PPR proteins are recognized by a degenerate 35 amino-acid motif folding into two antiparallel helices connected by a short loop/turn [28,29]. In association with the complexity of plant mitochondria gene expression, PPR proteins have been shown to play multifarious functions in organellar RNA metabolism, such as RNA stability and protection [12,30], C-to-U RNA editing [13], mRNA translation [31,32,33], and group II intron splicing [11,12,34,35,36]. Members of the PPR family are also linked to fertility restoration, where they regulate the expression of mitochondrial CMS-associated ORFs [37,38].

Genetic and biochemical studies indicated that PPRs are sequence-specific RNA-binding trans-factors, and that RNA recognition is mostly mediated by amino acids found at positions 5 and 35 in each PPR repeat. Association with each of the four RNA bases involves specific amino-acid combinations that are the basis of the PPR-RNA recognition code [39,40]. These data were further supported by the analysis of PPR protein-RNA crystal structures [28,29,41,42,43,44]. PPRs are classified into two main groups: P and PLS-type proteins, which in addition to canonical 35-amino acid PPR motifs (P) include long (L) or short (S) repeat variants [26,45]. While PLS-type proteins are almost exclusively associated with RNA editing [13,46], P-type PPR factors facilitate a wide range of organellar RNA expression steps going from stabilization to translation [34,47]. In this work, we analyzed the function of a mitochondrial P-type PPR factor that we named *MITOCHONDRIAL INTRON SPLICING FACTOR* 2 (*MISF2*), which is related to the PPR protein EMP10 in *Zea mays* [48]. As its ortholog in maize, the functions of MISF2 are essential for early embryo development. Embryo rescue techniques were used to investigate the molecular functions associated with two independent homozygous T-DNA insertional lines in *MISF2*. Loss-of-function mutants for *MISF2* are strongly affected in the splicing of the first intron in *nad2* gene (*nad2* intron 1), which encodes a ~55 kDa core subunits of CI. Accordingly, the biogenesis of the respiratory CI is strongly affected in the *misf2* mutants, while the transformation of the *MISF2* gene restored the wild-type phenotype and the mtRNA metabolism defects detected in the homozygous mutants. The conserved molecular functions between *MISF2* (in *Arabidopsis*) and *EMP10* (in maize, [48]) suggest that the common ancestor *MISF2/EMP10* was recruited to function in *nad2* intron 1 splicing prior to the divergence of monocot and dicot plants, i.e., about ~200 million years ago [49].

## 2. Results

### 2.1. The Topology of MISF2 Protein

**Figure 1:**
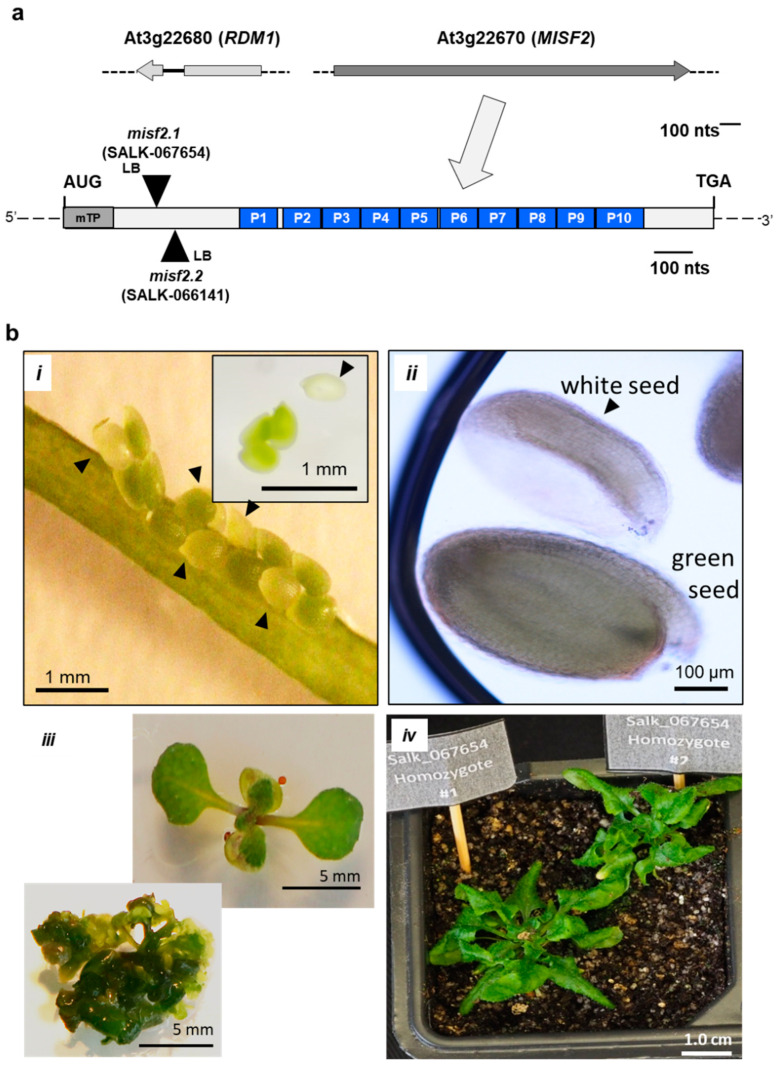
*MISF2* (At3g22670) gene topology and *misf2* mutant phenotypes. (**a**) Scheme of the At3g22670 locus and gene structure. The large arrow points toward motif-structure arrangements of the MISF2 coding region. The position of the two T-DNA insertion sites in the coding region of *MISF2* (i.e., SALK-line 067654, *misf2.1*, SALK-line 066141, and *misf2.2*) are located 324 and 350 nucleotides downstream to the ATG start codon, in a region that corresponds to the N-terminal domain of MISF2, upstream of the PPR motifs. (**b**) Morphologies of *misf2* hetero- and homozygous mutants. Green and white seeds harboring wild-type/heterozygous and homozygous mutant embryos respectively were collected from surface-sterilized immature siliques of heterozygous *misf2* plants (**i**) and sown on MS agar media supplemented with vitamins. Arrows point toward white seeds. Panel B (**ii**) shows differential interference contrast microscopy images (i.e., Nomarski) of embryos found in green or white seeds. Following germination, a few rescued homozygous *misf2* plantlets (**iii**) were able to survive on soil, although failed to set flowers and viable seeds (**iv**).

To better understand processes associated with mitochondrial RNA (mt-RNA) expression in plants, we assembled a collection of Arabidopsis T-DNA mutants affected in genes encoding mitochondria-targeted P-type PPR proteins and identified that heterozygous plants carrying insertions in the At3g22670 gene could not set homozygous mutants in their progeny. Domain search analysis using the PPR finder [50], PPRCODE [40], SMART [51] and CDD [52] algorithms indicated that the deduced product of *AT3G22670* gene (Figure 1, Figure S1) encodes a 562 amino-acid PPR protein with a predicted topology of NH_2_-165-P-3-P-P-P-P-P-P-P-P-P-42-COOH (where ‘P’ designates P-type PPR motifs and amino acids not assigned to any defined domain are specified by numbers) (Figure 1 and Figure S1a).

Subcellular localization prediction algorithms, available at the ExPASy portal (https://www.expasy.org; accessed on 28 January 2022), UniProt [53] and the ‘SUBcellular location database for Arabidopsis proteins’ (SUBA4, http://suba.live; accessed on 28 January 2022) [54], indicated the presence of a predicted 24-amino acid mitochondrial targeting sequence in the N-terminal region of MISF2 (Figure S1a). In silico 3D structure prediction, using the AlphaFold server [55], suggested that MISF2 harbors a typical PPR helical fold (Figure S1a), with an inner basic core representing the RNA binding surface, as previously indicated from the analysis of the plant PPR10 protein [29].

### 2.2. MISF2 Encodes a Lowly-Expressed P-Type PPR Protein That Is Localized in Mitochondria

**Figure 2:**
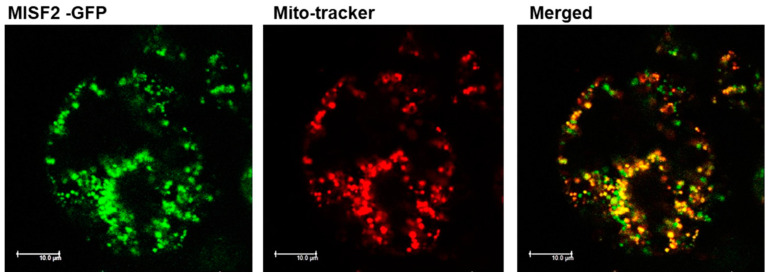
MISF2 is localized to the mitochondria. *Arabidopsis* plant cells were transformed with a construct expressing the GFP fused in frame to the N-terminal region (i.e., 203 amino acids) of the MISF2 protein. The fluorescence corresponding to the GFP (green, left), the MitoTracker^®^ marker (red, center), and the merged signals (right) are shown. Bars = 10 μm.

Expression analysis of *MISF2* was performed using publicly available microarray and high-throughput sequencing databases. The Arabidopsis Information Resource (TAIR) (http://www.Arabidopsis.org; accessed on 28 January 2022) (Figure S2a) and ‘Genevestigator analysis toolbox’ [56] (Figure S2b) databases indicated differential expression of the *MISF2* gene throughout development, with *MISF2* expression being dominant in embryonic organs, young developing leaves, apical root tissues, flowers, and the shoot apex. To further investigate the intracellular location of MISF2, a fragment comprising the first 203 amino acids of MISF2 was fused in-frame to GFP (MISF2-GFP) expressed in *Arabidopsis* cells and the subcellular localization of the resulting fluorescence examined by confocal microscopy (Figure 2). In agreement with the in silico data, the MISF2-GFP signal was detected as round-shaped particles that co-localized with those of the MitoTracker^®^ marker, a mitochondrion-specific fluorescent probe (Figure 2). These results are consistent with the predicted mitochondrial targeting of MISF2.

### 2.3. MISF2 Functions Are Required for Early Embryo Development in Arabidopsis thaliana

Several T-DNA insertion lines were identified within the *MISF2* gene. These include two independent lines: SALK_067654 (*misf2.1*) and SALK_066141 (*misf2.2*), which contain T-DNA insertions located 324 and 350 nucleotides downstream of *MISF2* translational start, respectively (Figure 1a and Figure S3a). Yet, no homozygous mutant plants could be recovered from the progeny of heterozygote *misf2* lines, suggesting that the At3g22670 gene product is essential for embryogenesis. The heterozygous *misf2.1* and *misf2.2* plant lines did not show any obvious phenotypes under normal growth conditions (see Section 4, Material and Methods), suggesting that homozygous mutants could be embryonically lethal. To test this assumption, we compared the developmental phenotypes of embryos contained in immature seeds (10 days after pollination) of heterozygous *misf2* with wild-type plants. Siliques of heterozygous *misf2* plants contained about one-quarter of yellow to white seeds (Figure 1(bi)), which later degenerated into shrunken and brown mature seeds. Microscopy analyses further indicated that green seeds in siliques of heterozygous *misf2* plants contained fully developed embryos, while white seeds had embryos arrested at the late torpedo/walking stick stages (Figure 1(bi,ii)).

### 2.4. Production of Embryo-Rescued misf2 Mutant Plants

Although *misf2* is not found among the 32 *Arabidopsis* embryo-defective *ppr* mutants of the ‘SeedGene’ database [57], our genetic and microscopic analyses indicate that *MISF2* is essential for proper embryo development (Figure 1b). Embryo rescue by in vitro culture allows to establish certain Arabidopsis mutants showing germination-defective phenotypes [58]. Among these are a few mutants affected in mitochondria biogenesis and function, such as the *cod1* [59], *ndufv1* [60] *cal1*/*cal2* [61,62], *nmat3* [63], or *rfl8* [33] mutants. Therefore, white seeds contained in young siliques of heterozygous *misf2* plants (i.e., 10~12 days post-anthesis, DPA), were sown on MS-agar plates supplemented with 1% sucrose and various vitamins (see Section 4, Materials and Methods) and then transferred to a controlled growth chamber. Indeed, under these conditions, 30% of the white seeds germinated after 3 months of culture and were then transferred to liquid culture using the same medium (see Section 4, Materials and Methods). PCR genotyping indicated that while green seeds derived from *misf2.1* or *misf2.2* heterozygote plants were either wild-type or heterozygous for the mutations, plantlets obtained from white seeds were all homozygous for either of the two *misf2* mutant alleles.

The conditions used to rescue homozygous *misf2.1* and *misf2.2* seedlings were similar to those reported for the embryo rescue of *Arabidopsis nmat3* [63] or *cod1* mutant [59]. Phenotypical variations between individual homozygous-rescued *misf2* plantlets were visible, with certain seedlings developing into slow-growing normal-looking plants with twisted leaves (Figure 1(biii)), while others produce miniature bushy-like structures (Figure 1(biii)). Similar observations were previously reported for several other *emb* mutants [64] affected in mitochondria biogenesis, including the rescued *nmat3* or *cod1* mutants [9,59,63]. A few homozygous-rescued *misf2* plantlets (e.g., Figure 1(biv)) could be further transferred and cultivated on soil, but none of the plants could produce viable seeds.

### 2.5. MISF2 Is Essential for nad2 Pre-mRNAs Processing in Arabidopsis Mitochondria

For RNA and protein analyses, we used 3-week-old MS-grown homozygous *misf2* mutant plantlets [64]. To further support the specific roles of MISF2 in mitochondria biogenesis, we also generated a functionally ‘complemented’ line (*misf2.2*/*MISF2*) by expressing the native *MISF2* gene in homozygous *misf2.2* plants (Figure S3b). Importantly, the expression of MISF2 in *misf2.2* plants restored the growth and developmental defect phenotypes associated with the *misf2.2* mutation. The complemented *misf2.2*/*MISF2* mutant plants were able to complete their life cycle and set viable seeds (Figure S3c).

**Figure 3:**
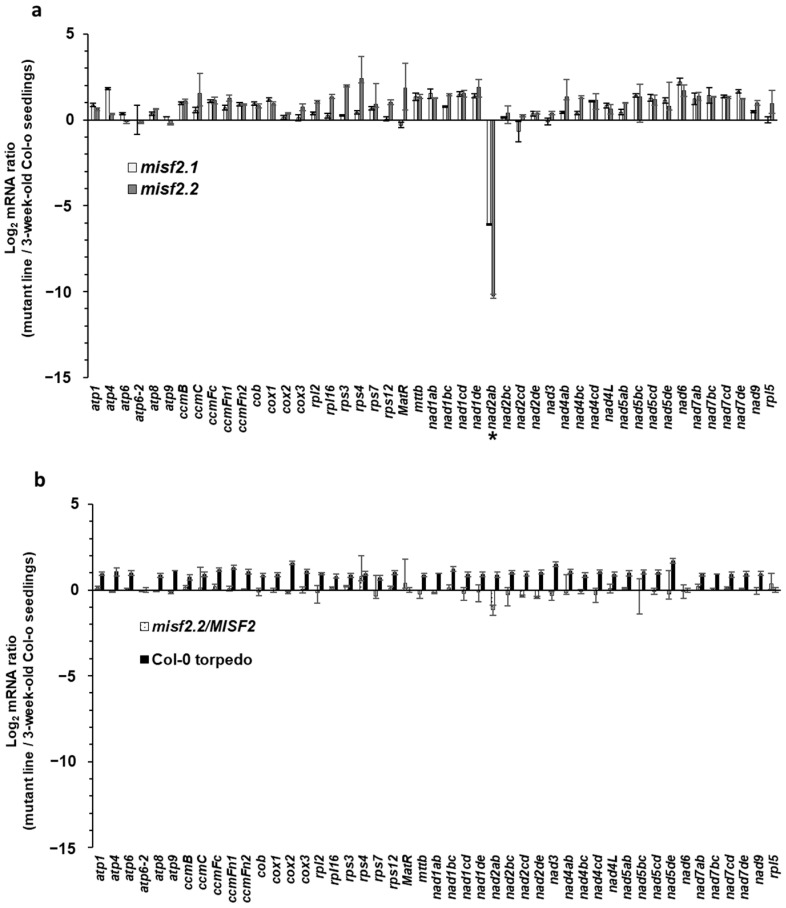
Relative accumulation of mitochondrial mRNAs in *misf2* mutants. Analysis of various mt-RNAs levels in *Arabidopsis* wild-type (Col-0), *misf2* mutants, and complemented *misf2.2*/*MISF2* plants by RT-qPCR. RNA extracted from 3-week-old wild-type seedlings (Col-0), 4-months-old rescued *misf2* mutants, plantlets derived from immature Col-0 seeds (i.e., at the torpedo stage), and functionally complemented *misf2.2*/*MISF2* mutants were reverse-transcribed and the relative steady-state levels of cDNAs corresponding to mitochondrial mRNAs evaluated by qPCR. Log_2_ ratios of mt mRNA abundances in *misf2.1* and *misf2.2* mutant lines (**a**), plantlets derived from immature Col-0 seeds, and complementation line (**b**) to those of 3-week-old MS-agar grown wild-type plants are shown. Asterisk indicates to reduced *nad2ab* transcript. The values are means of three biological replicates (error bars indicate one standard deviation).

The steady-state levels of mitochondrial mRNAs in homozygous (embryo-rescued) *misf2.1*, *misf2.2* and complemented *misf2.2*/*MISF2* mutants were analyzed by RT-qPCR in comparison with wild-type (Col-0) plants. This analysis revealed a strong reduction (i.e., about 70 to 1200 folds) in the accumulation of mature *nad2* transcripts spliced from their first intron in *misf2.1* and *misf2.2* mutants, respectively (i.e., *nad2* exons a and b (*nad2ab*), Figure 3a). The steady-state levels of most other mitochondrial transcripts, including *nad2* transcripts spliced from their other introns, were found to over-accumulate from 2 to 5 folds in both *misf2* mutant lines (Figure 3a). As a control, we also analyzed the RNA profiles of plantlets derived from immature wild-type embryos (from the heart to torpedo stage) that were grown under the same conditions as the rescued *misf2* mutants. The seedlings obtained from wild-type embryos did not show any significant reductions in the accumulation of mitochondrial transcripts, including *nad2* (Figure 3b). Similarly, the accumulation of *nad2* transcripts in functionally complemented plants were globally equivalent to those in wild-type plants (Figure 3b and Figure S3b). Based on these data, we concluded that the maturation defects observed for *nad2* transcripts in *misf2.1* and *misf2.2* plants relate to the functions of MISF2 and not to physiological differences between the embryo-rescued plantlets and 3-week-old Arabidopsis seedlings germinated on MS-media plates.

### 2.6. MISF2 Is Required for Efficient Splicing of nad2 Intron 1

**Figure 4:**
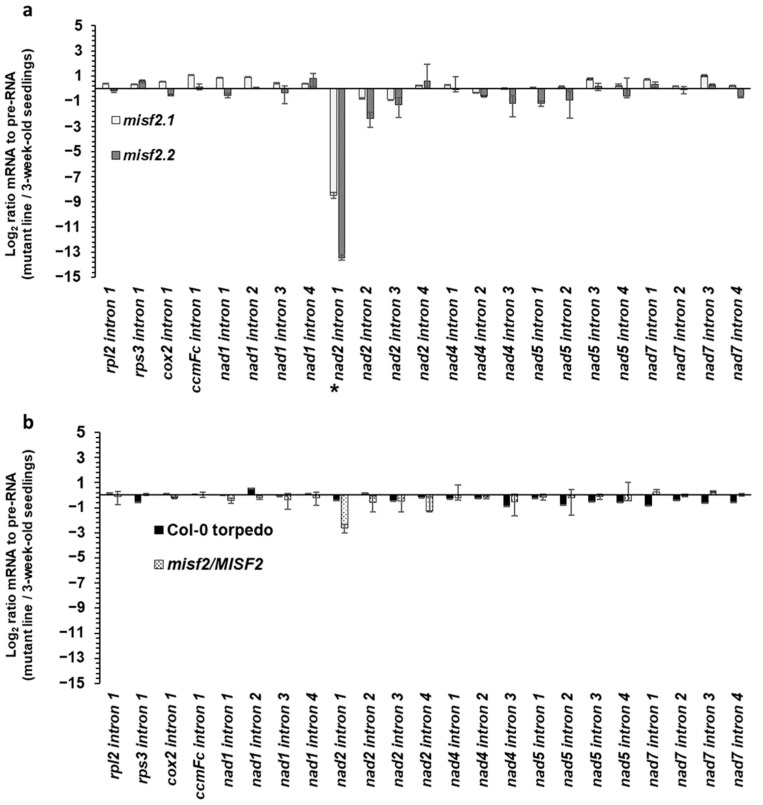
Mitochondrial intron splicing efficiencies in *misf2* mutants. The relative accumulation of mRNA and pre-RNA transcripts in wild-type, *misf2* mutants, and complemented *misf2.2*/*MISF2* plants, corresponding to the 23 group II intron sequences in *Arabidopsis*, was evaluated by RT-qPCR. The histogram shows the splicing efficiencies as indicated by the log_2_ ratios of pre-RNA to mature mRNA transcript abundance in *misf2.1* and *misf2.2* mutant lines compared with those in wild-type plants (**a**), as well as germinated wild-type seeds collected at the torpedo stage (Col-0-torpedo), and complemented line compared with those of wild-type plants (**b**). Asterisk indicates the altered splicing of *nad2* intron 1. The values are means of three (*misf2.1*, *misf2*.2/*MISF2*) and five (*misf2.2*, *Col-0*) biological replicates (error bars indicate one standard deviation).

We reasoned that the reduced steady-state levels observed for the upstream region of mature *nad2* transcripts (i.e., spliced exons ‘a’ and ‘b’) in the homozygous *misf2* mutants likely relate to defects in the excision of the first intron in *nad2*. We thus determined the splicing efficiencies of *nad2* intron 1 and that of the other 22 mitochondrial introns in wild-type plants and germinated embryos, as well as in *misf2* mutants and functionally complemented *misf2.2* plants by RT-qPCR. The obtained data revealed a strong reduction in the splicing efficiency of *nad2* intron 1, with splicing reductions reaching about 360 and 11,000 folds in *misf2.1* and *misf2.2* plants, respectively, compared with the wild type (Figure 4a).

In contrast to *nad2* intron 1, the splicing efficiency of other mitochondrial transcripts was not significantly affected in the homozygous *misf2* mutants, although small reductions (i.e., from 2.5 to 6.7 folds) in the splicing efficiencies of *nad2* introns 2 and 3 were seen in *misf2.2* plants. The reduction in *nad2* intron 1 splicing observed in *misf2* mutants was largely corrected in complemented *misf2.2* plants expressing the native *MISF2* gene (*misf2.2*/*MISF2*), strongly supporting the role of MISF2 in the processing of *nad2* intron 1 pre-mRNA (Figure 4b).

### 2.7. The MISF2 Protein Associates with nad2 Intron 1 In Vivo

**Figure 5:**
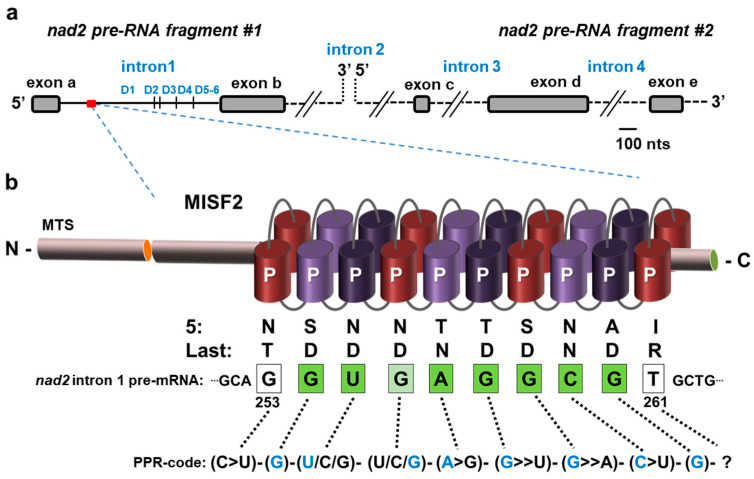
The predicted MISF2 binding site in *nad2* intron 1. (**a**) The expression of *nad2* in *Arabidopsis* mitochondria involves the transcription of two precursor RNA transcripts, which are divided by the second intron. The maturation of *nad2* requires the splicing of four introns found in *cis* (introns 1, 3, and 4) or *trans* (intron 2) configurations. The first pre-mRNA fragment consists of two exons separated by intron 1, while the second fragment harbors three exons separated by introns 3 and 4. The six typical stem-loop domains (D1–D6) are indicated for *nad2* intron 1. (**b**) MISF2 is a P-type PPR protein, which harbors a mitochondrial targeting sequence (MTS) and 10 PPR motifs. The fifth and the last amino acids of each PPR repeat (Figure S1) are indicated below each PPR repeats. The best corresponding RNA binding site (i.e., 5′-GUGAGGCG-3′) is indicated within the first intron of *nad2* pre-RNA fragment #1, with bases marked in green for perfect matches to the proposed binding site, in pale green for partial matches, and white for non-matching or unassigned nucleotides.

A scheme of *nad2* transcripts indicating the six typical stem-loop domains (D1–D6) within *nad2* intron 1 (*nad2* intron 1) is indicated in Figure 5a. PPR proteins are known to be sequence-specific RNA-binding factors [28,34,39,65,66,67]. A combinatorial code for RNA-recognition by PPR proteins was proposed, based on combinations of amino acids found at positions 5 and 35 of each PPR repeat [39,67,68]. The code applied to the 10 PPR repeats of MISF2 (Figure 1 and Figure S1a) indicated the following sequence: 5′-(C>U)-(G)-(U/C/G)-(U/C/G)-(A>G)-(G>>U)-(G>>A)-(C>U)-(G)-(?)-3′ (Figure 5b). Atomic structural model of MISF2 (Figure S1b) was predicted by the AlphaFold server [55]. A BLAST search along the updated *Arabidopsis* mtDNA (BK010421) revealed an eight-nucleotide matching sequence within the D1 stem-loop of *nad2* intron 1 (Figure 5b).

No other sequences of 10 bases long corresponding to the predicted MISF2 binding site could be identified elsewhere in the plant mitogenome. A model for the association of MISF2 with its predicted RNA binding site within *nad2* intron 1 is illustrated in Figure 5b. The in silico data, therefore, correlated with the ‘genetically defined’ RNA target of MISF2, *nad2* intron 1 (Figure 4 and Figure 5).

**Figure 6:**
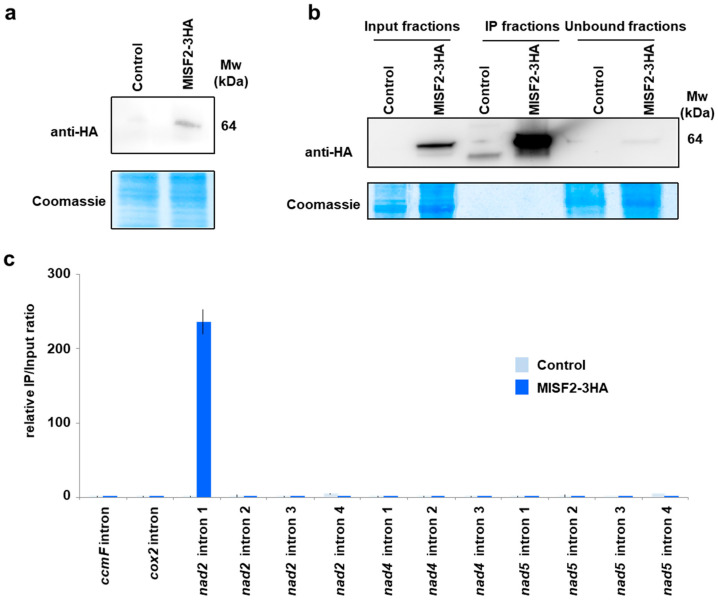
The MISF2 protein associates with *nad2* intron 1 in vivo. (**a**) Immunodetection of the MISF2-3HA fusion protein in protein extracts prepared form untransformed (control) and transformed (MISF2-3HA) *Arabidopsis* cell cultures. (**b**) Immunoprecipitation assays were conducted with the anti-HA antibody and the shown immunoblot analysis attests for the strong enrichment of the MISF2-3HA fusion in the immunoprecipitated (IP) fraction derived from the *Arabidopsis* transgenic cell line expressing the fusion. The weak MISF2-3HA signal in the unbound fraction demonstrates the efficiency of the immunoprecipitation. Parts of the blots stained with Coomassie blue are shown to display equal loading between samples. (**c**) Co-immunoprecipitated RNAs were analyzed by qRT-PCR using primer pairs specific to the indicated mitochondrial introns and relative enrichment ratios (immunoprecipitation fraction/input fraction) are shown.

To further examine the in vivo RNA targets of MISF2, a cell line expressing an HA-tagged version of MISF2 was produced. After confirming the expression of the tagged protein in vivo (Figure 6a), the MISF2-3HA protein was immunoprecipitated from total extracts (Figure 6b) and co-purified RNAs were analyzed by RT-qPCR (Figure 6c).

Primers amplifying *nad2* intron 1 were used in this analysis, along with other primers pairs targeting introns whose splicing was found to be slightly reduced in *misf2* plants, plus a few additional controls. The obtained results reveal a very strong co-enrichment of *nad2* intron 1, specifically in the co-IP ribonucleoprotein particle of MISF2-3HA. None of the other tested introns (i.e., the single introns within *ccmF* or *cox2* mRNAs, *nad2* introns 2, 3, and 4, *nad4* introns 1 to 3, or *nad5* introns 1 to 4) were co-enriched with MISF2-3HA, strongly supporting that *nad2* intron 1 is the in vivo RNA target of this PPR protein, thereby confirming that MISF2 specifically associates with its genetically defined intron RNA.

### 2.8. Analysis of the Respiratory Chain Biogenesis in misf2 Mutants

**Figure 7:**
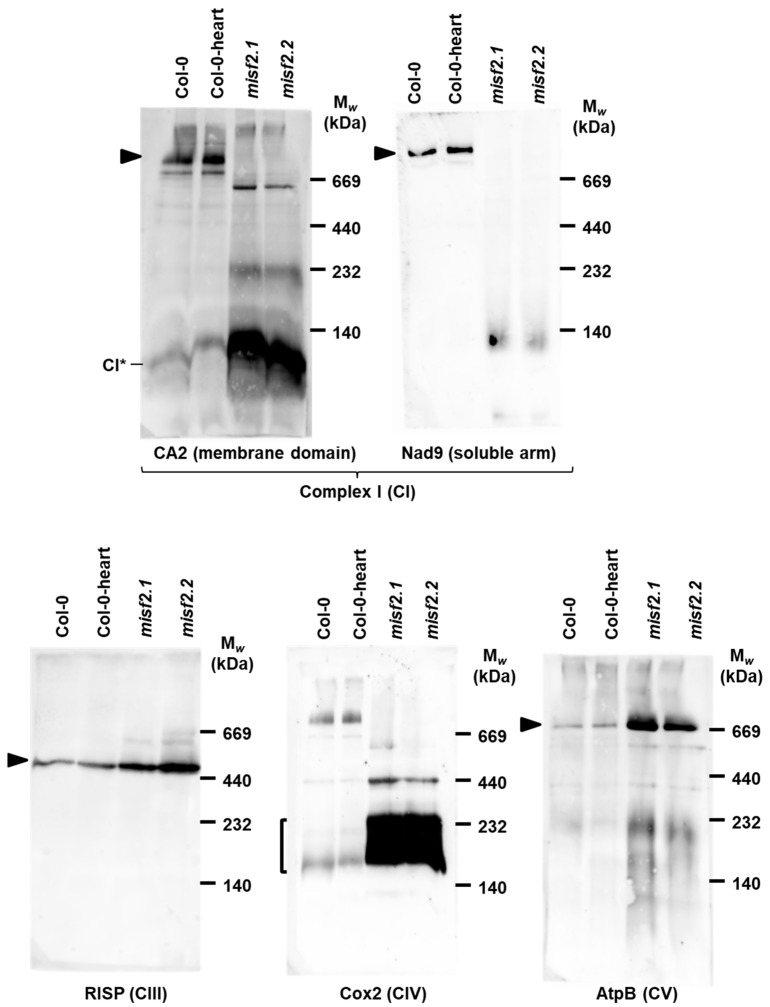
Holo-complex I is below detectable levels in *misf2* mutants. Blue native (BN)-PAGE analysis of crude organellar fractions was performed as described by [79]. Aliquots, equivalent to 40 mg of crude organellar membrane extracts, obtained from wild-type and *misf2* plants, were solubilized with digitonin and resolved by BN-PAGE. For immunodetection, the proteins were transferred onto PVDF membranes and probed with the antibodies indicated below each blot (Table S2). Arrows point toward native complexes I (~1000 kDa), CIII dimer (III_2_, ~500 kDa), CIV (about 200 and 220 kDa forms), and CV (~660 kDa) [69]. CI* indicates the ~85 kDa sub-CI assembly intermediate [5].

The respiratory system of plant cells is made of five major protein complexes, termed as complex I (CI, about 1000 kDa in size), CII (160 kD), dimeric complex III (III_2_, 500 kDa), CIV (200 and 220 kDa forms), and the ATP synthase (CV, 660 kDa) [69]. Plant mitochondria also harbor various enzymes that belong to the ‘alternative electron transport’ pathway, involving alternative NADH dehydrogenases and the alternative cytochrome oxidase [70]. Genetic and biochemical studies showed that Nad2 is essential for complex I (CI) biogenesis and function [5,71,72,73,74,75,76,77]. The reduction in *nad2* splicing (Figure 3 and Figure 4) suggests that the CI Nad2 subunit likely accumulates to very low levels in *misf2* plants. Indeed, BN-PAGE analysis of Arabidopsis respiratory complexes indicated that CI is below detectable levels in *misf2* mutant plants (Figure 7). Immunoblots made with antibodies against the carbonic anhydrase CA2 [78] further indicated the accumulation of several complex I assembly intermediates of about 610, 230 and 85 kDa in both *misf2* mutants. While CI was considerably reduced in both *misf2* mutants, BN-PAGE analyses indicated that other respiratory complexes, including CIII, CV, and particularly CIV, were rather upregulated in *misf2* plants (Figure 7).

**Figure 8:**
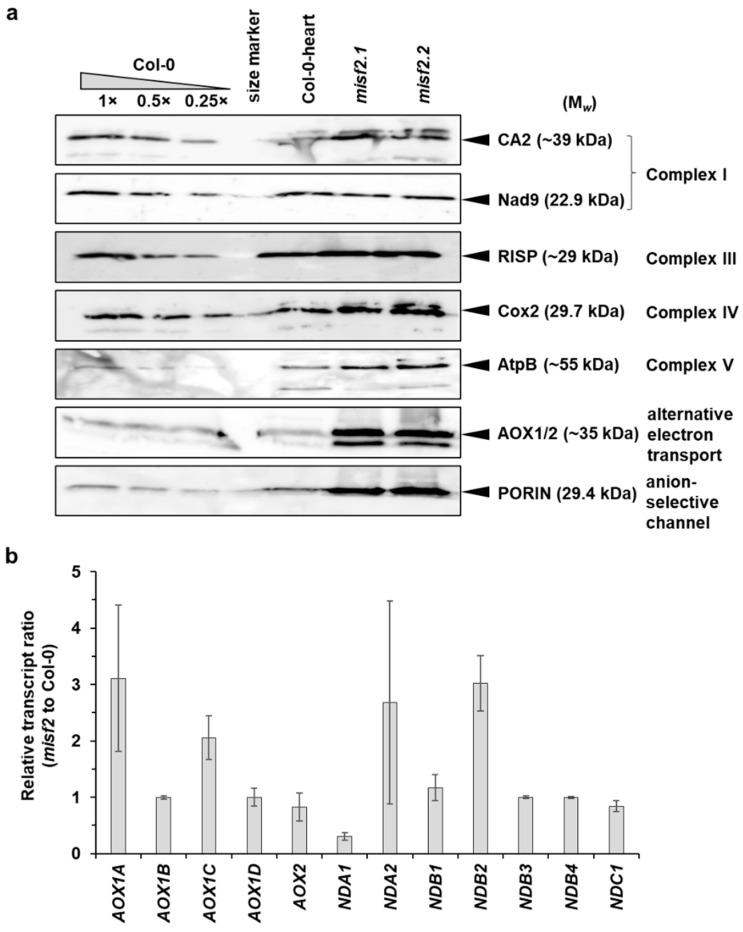
Relative accumulation of different mitochondrial proteins and *AOX* or *ND* transcripts in wild-type and *misf2* plants. (**a**) Immunoblots made with crude organellar fractions (equivalent to ~40 mg FW) extracted from 3-week-old MS-grown wild-type plants, in vitro germinated wild-type embryos at the heart to torpedo stages (Col-0 torpedo), and homozygous *misf2* plantlets. The blots were probed with antibodies raised against the indicated mitochondrial proteins. (**b**) Analysis of the steady-state levels of various alternative oxidase (AOX) and rotenone-insensitive NAD(P)H dehydrogenase (ND) mRNAs by RT-qPCR. The histogram displays relative mRNAs levels in *misf2* plantlets to 3-week-old MS-grown wild-type plants.

We further analyzed the relative accumulation of various mitochondrial proteins in Col-0, *misf2* mutants and the functionally complemented *misf2.2*/*MISF2* line by immunoblotting analysis using various antibodies raised against different plant mitochondrial proteins. The data indicated that the CI-subunits CA2 and Nad9 accumulate in similar quantities in *misf2* and wild-type plants. The levels of various other mitochondrial proteins, including the Rieske iron-sulfur protein (RISP) of CIII, the Cox2 subunit of CIV, the AtpB subunit of CV, and the mitochondrial outer-membrane voltage-dependent anion channel (VDAC or PORIN) proteins, were upregulated in *misf2* mutants, as compared with wild-type plants (Figure 8a).

In contrast, the accumulation of all tested mitochondrial proteins was equivalent between the complemented line (*misf2.2*/*MISF2*) and wild-type plants (Figure S4).

*Arabidopsis* mutants affected in CI biogenesis undergo oxidative stress and often subsequently show a strong induction of the alternative respiratory pathways [10,60,62,77,80]. Accordingly, the relative accumulation of transcripts corresponding to various alternative oxidase (AOX) and rotenone-insensitive NAD(P)H dehydrogenase (NDs) mRNAs in *misf2* was generally higher than in wild-type plants (Figure 8b). Similarly, immunoblot assays indicated that the steady-state levels of AOX1 or AOX2 proteins were higher in *misf2* compared with the wild-type (Figure 8a and Figure S4).

## 3. Discussion

### 3.1. The MISF2 Gene Encodes a Mitochondria-Localized PPR Protein That Plays Essential Roles in Early Embryo-Development of Arabidopsis Plants

Mitochondria play key roles in energy metabolism and are thus vital organelle for plant life. During evolution, the mitochondrial genomes of land plants have undergone increased plasticity, showing substantial variations in genome size and structures and gene expression patterns between species (reviewed by e.g., [1]). Angiosperm mtDNAs are the largest and least gene-dense genomes among eukaryotes [2]. mRNA production and expression in land plant mitochondria involve extensive processing steps, which include endonucleolytic RNA cleavages, 5′ and 3′ mRNA trimming, extensive sequence editing and, relevantly to our study, the removal of intron (mostly group II-type) sequences that interrupt the coding regions of many mitochondrial genes (reviewed by e.g., [11]). These essential activities may serve as key control points of plant mitochondrial gene expression and are facilitated by numerous RNA binding cofactors [11,12].

In this study, we assigned a role to an Arabidopsis PPR protein, namely the MISF2 encoded by the *AT3G22670* gene-locus in mt-RNA metabolism (i.e., *nad2* maturation) and respiratory complex I assembly. *MISF2*, as its maize orthologue (i.e., EMP10 [48]), encodes a lowly expressed P-type PPR protein comprising 10 PPR motifs (Figure 1, Figures S2 and S3), which is located within the mitochondria (Figure 2). Figure S5 represents the sequence alignment [81,82] (Figure S5a) and phylogenetic analysis of MISF2/EMP10 paralogs in different angiosperms, including dicot (i.e., *Arabidopsis*, cauliflower, tobacco, and tomato) and monocot (i.e., barley, maize, rice, sorghum, and wheat) plant species (Figure S5b).

The Arabidopsis SeqViewer database (https://seqviewer.arabidopsis.org; accessed on 28 January 2022), which uses the outdated TIGR 4.0 version of the Arabidopsis genome, suggests that the 5′ untranslated region (UTR) of *RDM1* (At3g22680) may overlap with the coding sequence of *MISF2* (encoded on the opposite strand). Such different-strand overlapping of genes is especially untypical when considering the 2152 nucleotide-long 5′ UTR suggested for the *RDM1* gene by the SeqViewer database in the compact genome of *A. thaliana*. However, this occurrence is not supported by the updated TAIR10 genome assembly that indicates a 76-nt-long 5′ UTR for *RDM1* gene (Figures S6 and S7a). Likewise, the annotated *RDM1* genes in other Arabidopsis species (i.e., *A. lyrata* LOC9321583 or *A. suecica* As03g023650) also harbor 5′ UTRs of about 80 and 200 nts, respectively, that do not overlap with *MISF2*. Rapid amplification of cDNA ends (RACE, Figure S3a) and RNA-seq data [83] (Figure S6) further showed that *RDM1* harbors a 5′-UTR between 51 and 78 nucleotides long, which consequently does not extend to the *MISF2* gene. This was also apparent by RT-PCRs with oligonucleotides designed to regions up- or down-stream of the 5′-UTR of *RDM1* (Figure S7a,b), which further indicated that *RDM1* is normally expressed in *misf2* mutant plants (Figure S3b).

As for *EMP10* in maize, downregulation of *MISF2* expression results in premature arrest of *Arabidopsis* embryo development at the late torpedo stage (Figure 1b), whereas the function of RDM1 is regarded as non-essential for embryogenesis in *A. thaliana* plants [84]. Nevertheless, it was important to confirm that the developmental defect phenotypes and altered mt-RNA metabolism we see in *misf2* mutants result directly from the downregulation of *MISF2* expression. To this end, we analyzed the growth phenotypes (Figure S3c) and organellar RNA and protein profiles in a functionally complemented *misf2* line (*misf2*/*MISF2*). These analyses revealed that the expression of MISF2 restored the embryogenesis defects and altered growth phenotypes associated with *MISF2* gene disruption (Figure S3b) and that MISF2 is directly required for *nad2* RNA maturation and respiratory CI biogenesis (Figure 3, Figure 4 and Figure S4). Co-IPs indicated that the MISF2 protein is specifically associated with its genetically defined intron RNA target (i.e., *nad2* intron 1) in vivo.

### 3.2. MISF2 Is Required for the Splicing of nad2 Intron 1

Most mitochondrial introns in angiosperms are classified as group II type [16]. Model introns belonging to this class are large catalytic RNAs that are characterized by a conserved secondary structure consisting of six double-helical domains (D1 to D6), radiating from a central hub, with an internal ORF encoding a maturase in D4 [85,86]. The excision of group II introns in vivo in bacteria and in the organelles of eukaryotic cells requires the action of various RNA binding protein cofactors. In canonical group II introns, these at least include the maturase proteins (that are most often encoded by the introns themselves) [87]. In plant mitochondria, many additional proteinaceous splicing factors are required, which either derive from an ancient group of maturases [18], or from various other RNA binding cofactors that were recruited during evolution to facilitate mitochondrial intron splicing [17,88].

The PPR protein family is the largest RNA binding protein family known in plants, with about 400 to 600 members targeted to mitochondria or plastids [89]. PPR proteins bind their RNA substrates in a sequence specific manner and were shown to play pivotal roles in various aspects of posttranscriptional RNA processing, including the excision of group II introns in land plant organelles [11,13,34,47]. Here, we analyzed the molecular functions of the Arabidopsis MISF2 protein by characterizing loss-of-function mutants. As no homozygous mutant individuals could be identified among mature seeds of self-fertilized heterozygous *misf2* progenies, we used embryo rescue approaches [59,63] to generate homozygous mutant plant material, which allowed us to analyze the role of MISF2 in mitochondrial RNA metabolism.

Analysis of mitochondrial RNA profiles in wild-type and *misf2* plants showed a large reduction in the accumulation of mature *nad2* mRNA in both mutant lines (Figure 3). The RT-qPCR analyses further revealed a strong reduction in the splicing efficiency of *nad2* intron 1 in *misf2* plants (Figure 4). The most probable RNA-binding site for MISF2 protein (i.e., GUGAGGCG) resides within the D1 stem-loop of *nad2* intron 1 (Figure 5), which also corresponds to the genetic and biochemical RNA target of MISF2 (Figure 3, Figure 4 and Figure 6). In model group II introns, maturases were shown to bind with great affinity and specificity to their cognate intron-RNAs, in particular to regions of D1 and around the maturase coding sequences within the D4 stem-loop of canonical group II intron [90]. It will therefore be interesting to investigate whether sequence changes within plant *nad2* intron 1 were accompanied by the recruitment of the PPR MISF2 factor to facilitate its splicing, possibly to stabilize or nucleate *nad2* intron 1 folding into a catalytically active structure.

Taken together, our data provide strong evidence that MISF2 is specifically required for *nad2* intron 1 splicing and that this RNA processing step is essential for early embryogenesis in Arabidopsis.

### 3.3. Embryo Development and Complex I Biogenesis

The electron transport chain is made of four major multi-subunit protein complexes, denoted as CI to CIV. Plants also possess several enzymes corresponding to non-phosphorylating bypasses of the electron transport chain (ETC), namely the alternative oxidase (AOX) and several rotenone-insensitive NAD(P)H dehydrogenases (NDs) [71,91,92,93,94]. The biogenesis of respiratory CI in angiosperms involves the incorporation of ~50 different subunits encoded by both mitochondrial (i.e., *nad1*, *nad2*, *nad3*, *nad4*, *nad4l*, *nad5*, *nad6*, *nad7*, and *nad9*) and nuclear gene loci [95]. These are incorporated into two main different CI domains, consisting of a membrane domain and a matrix (or peripheral) arm [3,5,10,96].

Nad2 is a pivotal subunit of CI, that is suggested to be incorporated very early during the assembly of the membrane arm [3,4,5,97]. The early steps of CI biogenesis involve the production of an ~85 kDa assembly intermediate of the membrane arm, which contains various gamma-type carbonic anhydrase subunits. Subsequently, Nad2 and a few other subunits are incorporated to form a ~200 kDa membrane-bound CI assembly intermediate [5]. It is therefore anticipated that a strong reduction in Nad2 would interfere with the assembly of the CI membrane arm, and hence, with the biogenesis of the ~1.0 MDa holo-CI. Consequently, BN-PAGE analysis of wild-type and mutant plants revealed a major reduction in CI abundance in both *misf2* mutant lines (Figure 7). Immunoblot analysis with anti-CA2 antibodies further revealed the existence of various CI intermediates in *misf2* mutants, among which a major particle of about 85 kDa, which was also observed in the *abo5* mutant that is impaired in *nad2* expression [74]. The CI particles of higher mass (i.e., 230 kDa and 610 kDa) detected in the mutants may correspond to Nad2-deprived assembly intermediates that are less stable than the ~85 kDa particles [5].

It has been demonstrated that the severity of CI deficiency correlates with the gravity of the phenotypes displayed by corresponding plant mutants [10,60,77]. Severe CI mutants are impaired in the storage of essential nutrients but not in the mobilization of stored reserves [60]. Accordingly, mutants affected in β-oxidation, a metabolic process by which fatty acids are broken down by various tissues to produce energy, contain embryos that are typically arrested at earlier developmental stages compared with CI mutants [98]. Embryo maturation is often incomplete in various CI mutants, leading to the production of seeds with reduced reserves and germination capacity. One can anticipate that altered respiration interferes with numerous essential metabolic activities, resulting in altered embryo development.

In our study, we noticed that a severe defect in the production of the Nad2 subunit of CI results in impaired embryogenesis and a loss of germination capacity of Arabidopsis mutant seeds. However, most characterized plant CI mutants are generally able to germinate under standard culture conditions (see e.g., [21,76,77,99,100,101]). The inability of *misf2* mutants to germinate under normal conditions is expected to result from an early arrest of mutant embryo development, placing *misf2* mutants among the most severe CI mutants reported so far. We currently do not know the role that the embryo-rescue medium plays in improving the seed germination of *misf2* mutants. It may be due to the presence of certain important chemicals in the rescue medium, or simply to a weakening of the seed coat by the high sugar concentration of the medium. Once germination was induced, we could observe that *misf2* mutants often showed growth phenotypes such as other Arabidopsis CI mutants (Figure 1(biv)). It was previously suggested that once photosynthesis is established, growth is to a lesser extent dependent on the application of external vitamins and/or sugars [60]. Subsequently, rescued *misf2* mutants can slowly proceed with their vegetative growth phase but remain unable to complete their life cycle, flower, and produce viable seeds.

## 4. Materials and Methods

### 4.1. Plant Material and Growth Conditions

*Arabidopsis thaliana* of the Columbia (Col-0) accession was used in all experiments. The wild-type (Col-0 line), SALK-067654 (*misf2.1*), and SALK-066141 (*misf2.2*) mutants were obtained from the *Arabidopsis* Biological Resource Center (ABRC, Columbus, OH, USA). Prior to germination, seeds obtained from wild-type and mutant lines were surface sterilized with Cl_2_ gas, generated by the addition of 1 mL HCl per 50 mL of bleach (sodium hypochlorite 4.7%), for 4 h at room temperature (RT). The seeds were then sown on MS-agar plates containing 1% (*w*/*v*) sucrose or rescued by a method described in detail below. For synchronized germinations, the seeds were kept in the dark for 5 days at 4 °C and then grown under long-day condition (LD, 16:8-h) in a growth chamber (Percival Scientific, Perry, IA, USA) at 22 °C and under light intensity of 300 µE m^−2^ s^−1^. PCR was used for genotyping the plants using specific oligonucleotides listed in Table S1. Sequencing of specific PCR products was used to check the T-DNA insertion site in both mutant lines.

### 4.2. GFP Localization Assay

The DNA region encoding the first 203 amino acids of MISF2 was PCR amplified with specific oligonucleotides (i.e., *misf2*-B1 and *misf2*-B2; Table S1. The 609 nts PCR DNA fragment was cloned into the pDONR207 vector using the Gateway BP clonase enzyme mix and verified by Sanger sequencing. The entry clone was then transferred into the pGWB5 vector by Gateway LR reaction to create a GFP translational fusion between the MISF2 N-terminal sequence and the GFP coding sequence. The vector was transformed into *Agrobacterium tumefaciens* (strain C58C51) and used to transform *Arabidopsis* plant cells, as previously described [102]. Transgenic cells were selected on hygromycin and GFP fluorescence was visualized by confocal microscopy Leica TCS SP8. To visualize mitochondria in vivo, plant cells were incubated with 1 µM MitoTracker^®^ Red (Thermo Fisher, Scientific, Waltham, MA, USA) for 10 min at room temperature prior to observation under confocal microscopy.

### 4.3. Embryo-Rescue and Establishment of Homozygous misf2 Mutants

Siliques from wild-type and heterozygous *misf2* plants were surfaced sterilized with 6% bleach solution for 10 min at RT. The seeds were then soaked in a 70% ethanol solution for 10 min at RT, washed briefly with sterile DDW, and opened in a sterile hood. Green and white seeds obtained from siliques of heterozygous *misf2* plants 10 days after self-fertilization were sown on MS-agar plates supplemented with 1% (*w*/*v*) sucrose and 10 mg myoinositol, 100 μg thiamine, 100 μg pyridoxine, and 100 μg nicotinic acid. For DNA and RNA analysis, we used *Arabidopsis* wild-type and *misf2* plantlets at stage R6 (i.e., 6 to 8 leaves) [64]. To obtain larger quantities of plant material, plantlets at stage R6 were grown on MS-agar plates and then transferred to MS-based liquid medium supplemented with 1% (*w*/*v*) sucrose and 10 mg myoinositol 100 μg Thiamine, 100 μg Pyridoxine, and 100 μg nicotinic acid and incubated at 22 °C under a light intensity of 300 µE m^−2^ s^−1^ with moderate (50~100 RPM) shaking.

### 4.4. Functional Complementation—Establishment of misf2.2/MISF2 Plants

For the complementation assay, the *MISF2* gene and its predicted promoter region were amplified by PCR from *Arabidopsis thaliana* total DNA using the *MISF2*-promo-B1 and *MISF2*-Cpl-B2 primers, cloned into the pDONR207 vector by Gateway^®^ BP reaction (Invitrogen, Waltham, MA, USA), and subsequently transferred into the pGWB13 expression vector [103] by LR reaction (Invitrogen, Waltham, MA, USA). The resulting vector was used to transform *misf2* heterozygous plants by floral dip transformation. Transformed plants were selected on hygromycin and transgenic homozygous mutants were identified by PCR genotyping.

### 4.5. Expression of the 3XHA-Tagged MISF2 Protein in Arabidopsis Cell Cultures

For expressing a 3XHA-tagged version of MISF2 in *Arabidopsis* cell cultures, the MISF2 coding sequence was amplified by PCR using the MISF2-B1 and MISF2-Cpl-B2 primers, cloned into the pDONR207 vector by Gateway^®^ BP reaction (Invitrogen, Waltham, MA, USA), and subsequently transferred into the pGWB14 expression vector [103]. The resulting construct was used to transform the PSBD *Arabidopsis* cell line as previously described [102].

### 4.6. Microscopic Analyses of Arabidopsis Wild-Type and Mutant Plants

Analysis of whole plant morphology, roots, leaves, siliques, and seeds of wild-type and mutant lines were examined under Stereoscopic (dissecting) microscope or light microscope at the bio-imaging unit of the Institute of Life Sciences (The Hebrew University of Jerusalem, Jerusalem, Israel). Seeds were incubated with Hoyer solution for 30 min and the cleared samples were analyzed by differential interference contrast (Nomarski) microscopy.

### 4.7. RNA Extraction and Analysis

RNA extraction and analysis was performed essentially as previously described [21,23,104,105,106]. Total RNA was prepared from 200 mg seedlings grown on MS-agar plates supplemented with 1% sucrose using the RNAzol RT reagent (Sigma-Aldrich, St. Louis, MO, USA). The RNA was then treated with RNase-free DNase I prior to its use in the assays. RT-qPCR was performed with specific oligonucleotides designed to exon-exon (mRNAs) regions corresponding to mitochondrial genes and intron-exon regions (pre-mRNAs) within each of the 23 group II introns in *Arabidopsis thaliana* (Table S1). cDNA was synthesized by reverse transcription with the Superscript III reverse transcriptase (Invitrogen, Waltham, MA, USA), using 1–2 µg of total RNA and 250 ng of a mixture of random hexanucleotides (Promega, Mannheim, Germany) and incubated for 50 min at 50 °C. Reactions were stopped by 15 min incubation at 70 °C and the RT samples served directly for real-time PCR on a LightCycler 480 (Roche, Penzberg, Germany) using 2.5 μL of LightCycler 480 SYBR Green I Master mix and 2.5 μM of primers in a final volume of 5 µL. Reactions were performed in triplicate in the following conditions: pre-heating at 95 °C for 10 min followed by 40 cycles of 10 s at 95 °C, 10 s at 58 °C, and 10 s at 72 °C. The nucleus-encoded 18S rRNA (At3g41768) and the mitochondrial 26S ribosomal rRNA subunit (ArthMr001) were used as reference genes.

### 4.8. Rapid Amplification of Complementary End (RACE) Analyses

Poly-A+ cDNA libraries were obtained from total RNA extracted from 3-week-old MS-grown Arabidopsis plants, using the Dynabeads™ mRNA Purification Kit (Thermo-Fisher, Thermo Fisher, Kiryat Shmona, Israel). The 5’ and 3′ ends of RDM1 were established by RACE analysis, using the SMARTer^®^ RACE 5′/3′ Kit (Takara Bio Inc., Kusatsu, Shiga, Japan). For the analysis of the 5′ UTR of *MISF2*, we performed an ‘inverse single strand RACE’ analysis. First, a cDNA corresponding to *MISF2* mRNA was generated by RT-PCR with a primer phosphorylated by T4 Polinucleotide Kinase (Promega). The cDNA was self-ligated with T4 RNA Ligase (Promega, Mannheim, Germany) overnight at 25 °C. The 5′ end of the *MISF2* gene was generated by PCR with primers designed near the ends of the gene (i.e., *MISF2-RACE_S1* and *MISF2-RACE_AS2*) and analyzed by sequencing.

### 4.9. Crude Mitochondria Preparations

Crude mitochondria extracts were prepared essentially as described previously [79]. To this end, 200 mg of plantlets grown in liquid culture were harvested and homogenized in 2 mL of 75 mM MOPS-KOH, pH 7.6, 0.6 M sucrose, 4 mM EDTA, 0.2% polyvinylpyrrolidone-40, 8 mM L-cysteine, 0.2% bovine serum albumin, and protease inhibitor cocktail ‘complete Mini’ from Roche Diagnostics GmbH (Mannheim, Germany). The lysate was filtrated through one layer of Miracloth and centrifuged at 1300× *g* for 4 min at 4 °C (to remove cell debris). The supernatant was then centrifuged at 22,000× *g* for 10 min at 4 °C. The resulting pellet containing thylakoid and mitochondrial membranes were washed twice with 1 mL of wash buffer 37.5 mM MOPS-KOH, 0.3 M sucrose, and 2 mM EDTA, with pH 7.6 prior to use.

### 4.10. Blue Native PAGE Analysis of Respiratory Complexes

Blue native (BN)-PAGE of crude organellar membranous fractions was performed according to the method described in Ref. [79]. An aliquot equivalent to 40 mg of crude Arabidopsis mitochondria extracts was solubilized with 5% (*w*/*v*) digitonin in BN-solubilization buffer (30 mM HEPES, pH 7.4, 150 mM potassium acetate, 10% (*v*/*v*) glycerol) and then incubated on ice for 30 min. The samples were centrifuged for 8 min at 20,000× *g* to pellet non-solubilized material and 0.2% (*v*/*v*) of Serva Blue G was added to the supernatant. The samples were then loaded onto a native 4% to 16% linear gradient gel. For ‘non-denaturing-PAGE’ immunoblotting, the gel was transferred to a PVDF membrane (Bio-Rad) in Cathode buffer (50 mM Tricine and 15 mM Bis-Tris-HCl, pH 7.0) for 16 h at 4 °C at constant current of 40 mA. The blots where then incubated with antibodies against mitochondrial proteins (Table S2) and hybridization signals were identified by chemiluminescence assay after incubation with an appropriate horseradish peroxidase (HRP)-conjugated secondary antibody.

### 4.11. RNA Co-Immunoprecipitation Assays

Immunoprecipitation of MISF2-3HA were performed using the μMACS HA-Tagged Protein Isolation Kit (Miltenyi Biotec, Bergisch Gladbach, Germany) following a procedure previously described in Wang et al., 2020 [88].

## 5. Conclusions

Angiosperms encode numerous PPR proteins that are predominantly localized in plastids and mitochondria, which carry essential roles in organellar RNA metabolism. These include the EMP10 protein, which regulates the maturation of *nad2* in maize mitochondria [48]. Analysis of the protein and RNA profiles of mutants affected in the Arabidopsis orthologous gene, designated *MITOCHONDRIAL SPLICING FACTOR* 2 (*MISF2*, encoded by At3g22670 gene), indicates that MISF2 also functions specifically in the excision of the first intron of *nad2*. Plant mutants affected in *MISF2* accumulate high levels of *nad2* pre-RNA due to a strong defect in *nad2* intron 1 splicing. The altered splicing found in *misf2* (or *emp10*) is tightly associated with CI biogenesis defects and arrested embryonic development. Together, these data show that the molecular functions are conserved between the Arabidopsis MISF2 protein and its related EMP10 homolog in maize [48], which suggests that the common PPR ancestor of MISF2 and EMP10 has been recruited to act in *nad2* intron 1 splicing prior to the divergence of monocot and dicot plant species [49]. Our results provide important insights into the roles of nuclear-encoded PPR factors in mitochondria gene expression and the biogenesis of the respiratory system during early plant life.

## Data Availability

The data presented in this study are openly available in FigShare at 10.6084/m9.figshare.19244277, accessed on 28 January 2022.

## Supplementary Materials

The following supporting information can be downloaded at: https://www.mdpi.com/article/10.3390/ijms23052670/s1.
