# Supplementary material for: MISF2 Encodes an Essential Mitochondrial Splicing Cofactor Required for nad2 mRNA Processing and Embryo Development in Arabidopsis thaliana
Source: Int J Mol Sci. 2022 Feb 28;23(5):2670. doi: 10.3390/ijms23052670 (PMC8910670; doi:10.3390/ijms23052670)
Supplement: Supplementary file 1 [file ijms-23-02670-s001.zip › ijms-1566131-supplementary.pdf]

Supplementary Materials

MISF2 encodes an essential mitochondrial splicing cofactor required for *nad2* mRNA processing and embryo development in *Arabidopsis thaliana*

Tan-Trung Nguyen[1,†], Corinne Best[2,†], Sofia Shevtsov[2], Michal Zmudjak[2], Martine Quadrado[1], Ron Mizrahi[2], Hagit Zer[2], Hakim Mireau[1,*], Oren Ostersetzer-Biran[2,*]

[1] Affiliation 1; Institut Jean-Pierre Bourgin INRAE, AgroParisTech, CNRS, Université Paris-Saclay, Versailles, France
[2] Affiliation 2; Department of Plant and Environmental Sciences, The Alexander Silberman Institute of Life Sciences, The Hebrew University of Jerusalem, Givat-Ram, Jerusalem 91904, Israel.
† These authors have equally contributed to the MS.
* Correspondence: Hakim Mireau, E-mail, hakim.mireau@versailles.inra.fr, and Oren Ostersetzer-Biran. E-mail, oren.ostersetzer@mail.huji.ac.il.

Supplementary data files:

Figure S1: The topology and structure of MISF2 protein.
Figure S2: MISF2 gene expression pattern during Arabidopsis development.
Figure S3: Nucleotide sequence of the *MISF2* gene and precise location of T-DNA insertion sites.
Figure S4: Steady-state level analysis of various mitochondrial proteins in wild type, *misf2.2* and functionally complemented *misf2.2* plants.
Figure S5: Phylogenetic analysis of homologous MISF2 in plants.
Figure S6: Transcription profiles of *RDM1* gene by RNA-seq analyses
Figure S7: Analysis of the 5′ UTR of RDM1 transcript by RT-PCR.
Table S1: List of oligonucleotides used in this study.
Table S2: List of antibodies used in this study.

a

MPP/ICP55 cleavage site

MLTKLRISKLVSYTLPRRIFQRRFLVTNNTAEESPIVAAESPELPSWIKDFLSNKPSSSS
SSVSKDDEDFVIPSLANWVESQKFSRQQVSEGNVVKKPVEDIDKVCDFLNKKDTSHEDVV
KELSKCDVVVTESLVLQVLRRFSNGWNQAYGFFIWANSQTGYVHS|GHTYNAMVDVLGKC
RNFDLMWELVNEMNKNEESKL|VTI|LDTMSKVMRRLAKSGKYNKAVDAFLEMEKSYGVK
TD|TIAMNSLMDALVKENSIEHAHEVFLKLFDTIKPD|ARTFNILIHGFCKARKFDDARA
MMDLMKVTEFTPD|VVTYTSFVEAYCKEGDFRRVNEMLEEMRENGCNPN|VVTYTIVMHS
LGKSKQVAEALGVYEKMKEDGCVPD|AKFYSSLIHILSKTGRFKDAAEIFEDMTNQGVRR
D|VLVYNTMISAALHHSRDEMALRLLKRMEDEEGESCSPN|VETYAPLLKMCCHKKKMKL
LGILLHHMVKNDVSID|VSTYILLIRGLCMSGKVEEACLFFEEAVRKGMVPR|DSTCKML
VDELEKKNMAEAKLKIQSLVQSKTMIDSHSPLSVS

b

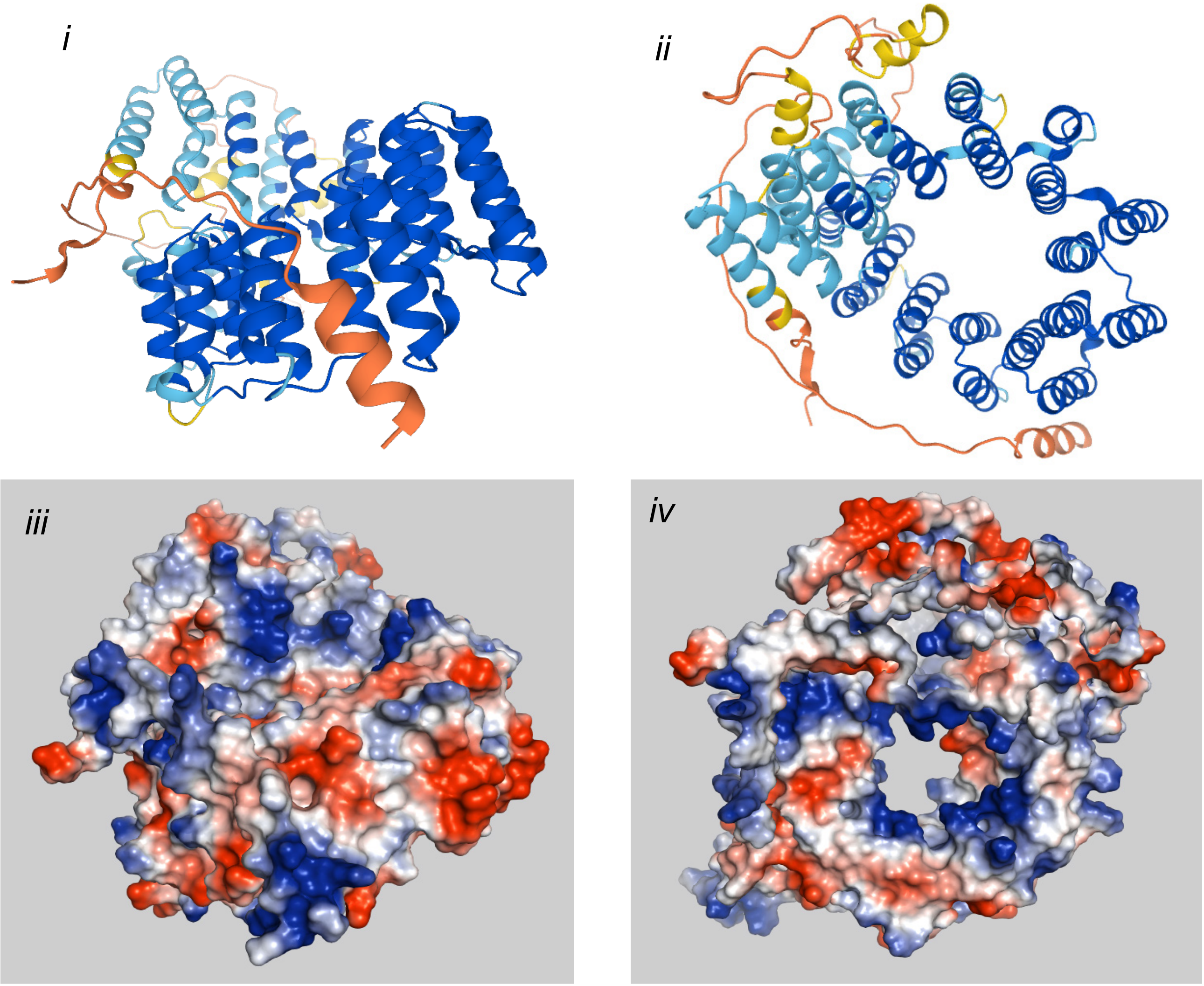

Figure S1. The topology and structure of MISF2 protein. (a) Amino acid sequence of the MISF2 protein (At3g22670). The region corresponding to the predicted mitochondrial targeting sequence is underlined and highlighted in red. The predicted cleavage sites of the Mitochondrial Processing Peptidase (MPP) or Intermediate Cleavage Peptidase 55 (ICP55) is indicated with a red arrow. The 10 PPR motifs (highlighted in blue letters) were predicted with the PPRfinder (Gutmann et al. 2020), PPRCODE (Shen et al. 2019), SMART (Letunic et al. 2012) and CDD (Marchler-Bauer et al. 2003) web-servers. Underlined letters indicate the 5 and 35 positions within each PPR repeat. (b) Atomic structural model of MISF2 predicted by the AlphaFold server (Jumper et al. 2021). Ribbon structures (i and ii) were generated by the PyMol software suite. Panels "iii" and "iv" represent charges on the surface of MISF2 from two different angles, with red, white, and blue for negatively, neutral, and positively charged residues, respectively.

a

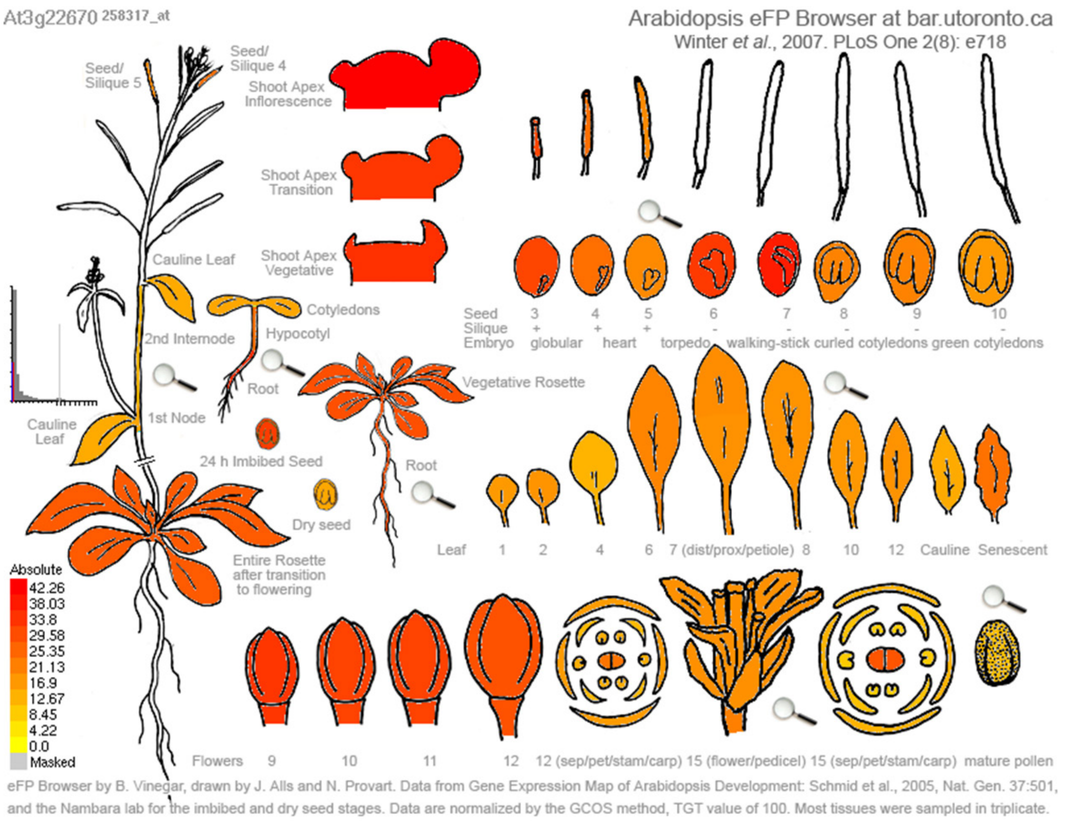

b

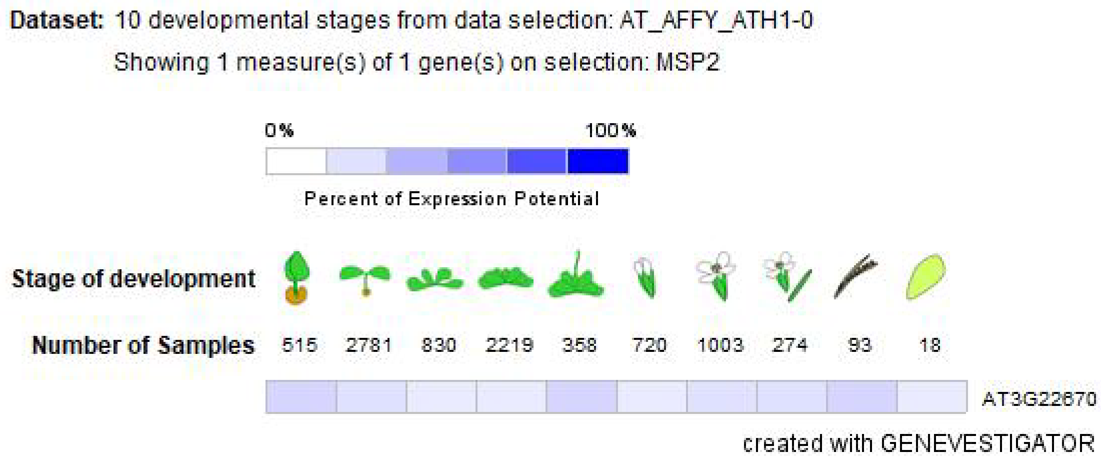

Figure S2. MISF2 gene expression pattern during Arabidopsis development. The expression pattern of MISF2 was analyzed using publicly available microarray and high-throughput RNAseq databases, including (a) 'The Arabidopsis Information Resource' (TAIR; http://www.arabidopsis.org), and (b) 'Genevestigator analysis toolbox' database (Hruz et al. 2008, Zimmermann et al. 2004).

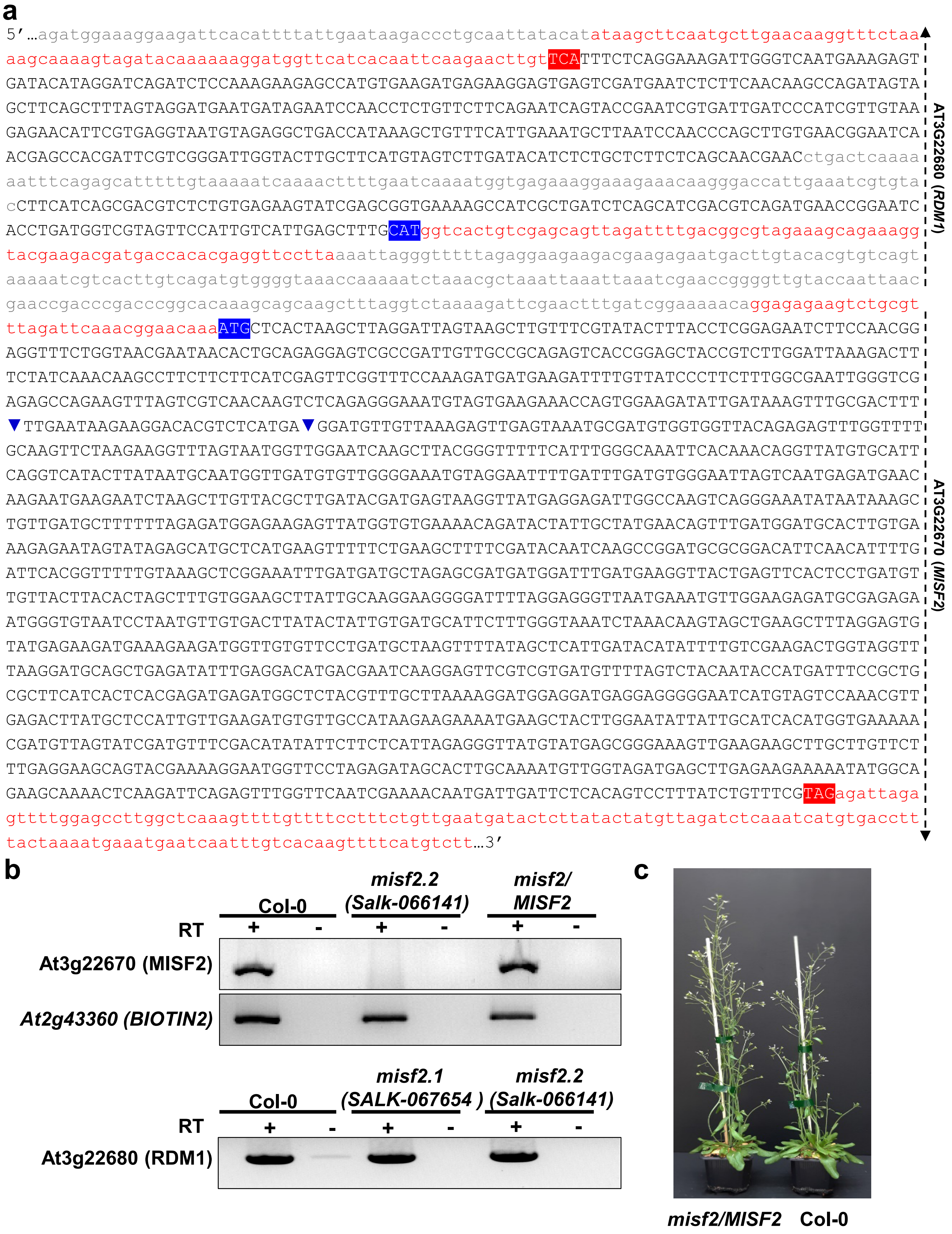

Figure S3. Nucleotide sequence of the MISF2 gene and precise location of T-DNA insertion sites. (a) Nucleotide sequence of MISF2 and RDM1, encoded by the At3g22670 and At3g22680 gene-loci, respectively. Lowercase letters colored in red indicate 5′ and 3′ untranslated regions (UTRs), as revealed by 5′ and 3′ RACE analysis, while uppercase letters correspond to the MISF2 open reading frame. Positions of T-DNA insertions in *misf2.1* (SALK-067654, 324 nts upstream to the AUG) and *misf2.2* (SALK-066141, 350 nts upstream to the AUG) lines are indicated. (b) RT-PCR analysis monitoring the expression of MISF2 in wild type (Col-0), homozygous *misf2.2* mutants (Salk_066141) and functionally complemented *misf2.2* plants (*misf2*/MISF2). (c) Photo of 5-week-old plants displaying the phenotypic recovery of *misf2.2* mutant by the expression of a complementary gene.

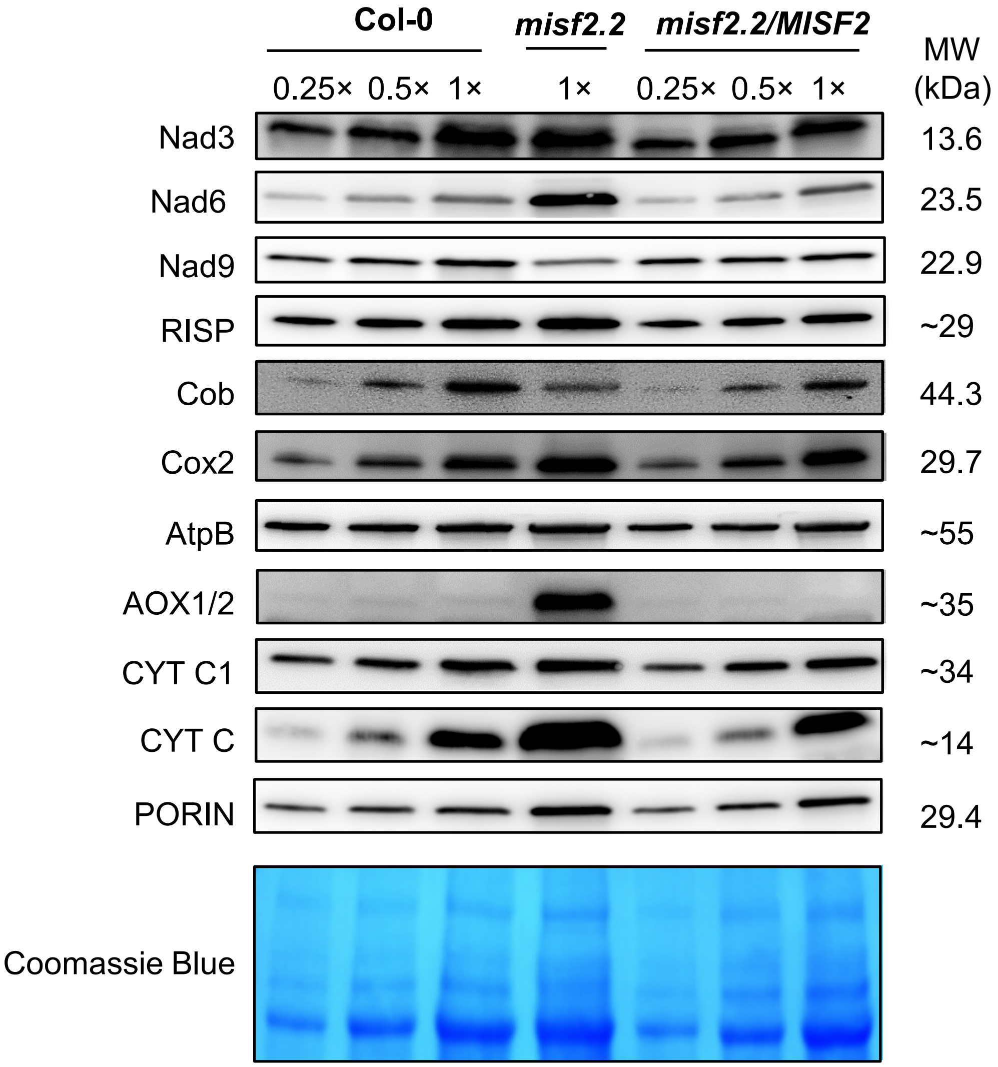

Figure S4. Steady-state level analysis of various mitochondrial proteins in wild type, *misf2.2* and functionally complemented *misf2.2* plants. Immunoblots performed with total proteins extracted from 3-week-old MS-grown wild type plants, rescued *misf2.1* plantlets, and complemented *misf2.2:35S-MISF2* plants. The blots were probed with polyclonal antibodies raised against the indicated mitochondrial proteins. Dilution series of proteins extracted from wild type and complemented plants were loaded for signal comparison with *misf2.1* extracts. Detection was carried out by chemiluminescence assays after incubation with HRP-conjugated secondary antibody. The original SDS-PAGE, following Coomassie-blue staining, is indicated below the immunoblot panels.

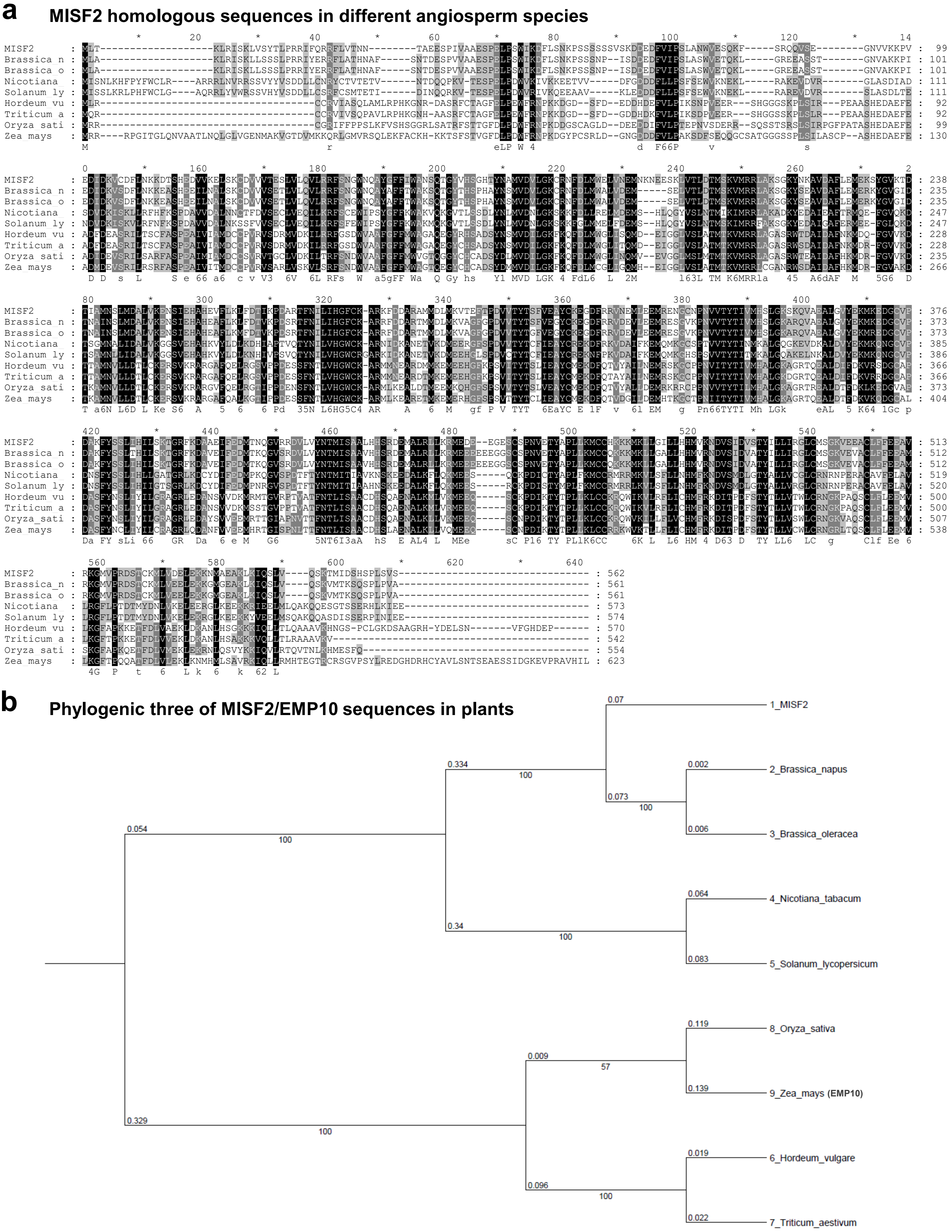

Figure S5. Phylogenetic analysis of homologous MISF2 in plants. Homologues sequences to the MISF2 protein were obtained by the Basic Local Alignment Search Tool (BLAST), against the plant genome sequence databases. (a) Alignment of homologous MISF2 proteins from *A. thaliana* (NP_188906.1), *B. napus* (XP_013736563.2), *B. oleracea* (XP_013623013.1), *N. tabacum* (XP_016433260.1), *S. lycopersicum* (XP_004247584.1), *H. vulgare* (XP_044948743.1), *T. aestivum* (XP_044382896.1), *O. sativa* (XP_015628161.1) and *Z. mays* (EMP10, NP_001183846.1), was conducted with the MAFFT multiple sequence alignment server [83] and displayed using GeneDoc [82] with the conserved residue shading mode. A phylogeny tree (b) was constructed, using the MAFFT and Archaeopteryx tree viewer (http://www.phylosoft.org/archaeopteryx) (bootstrap values for 1,000 replicates).

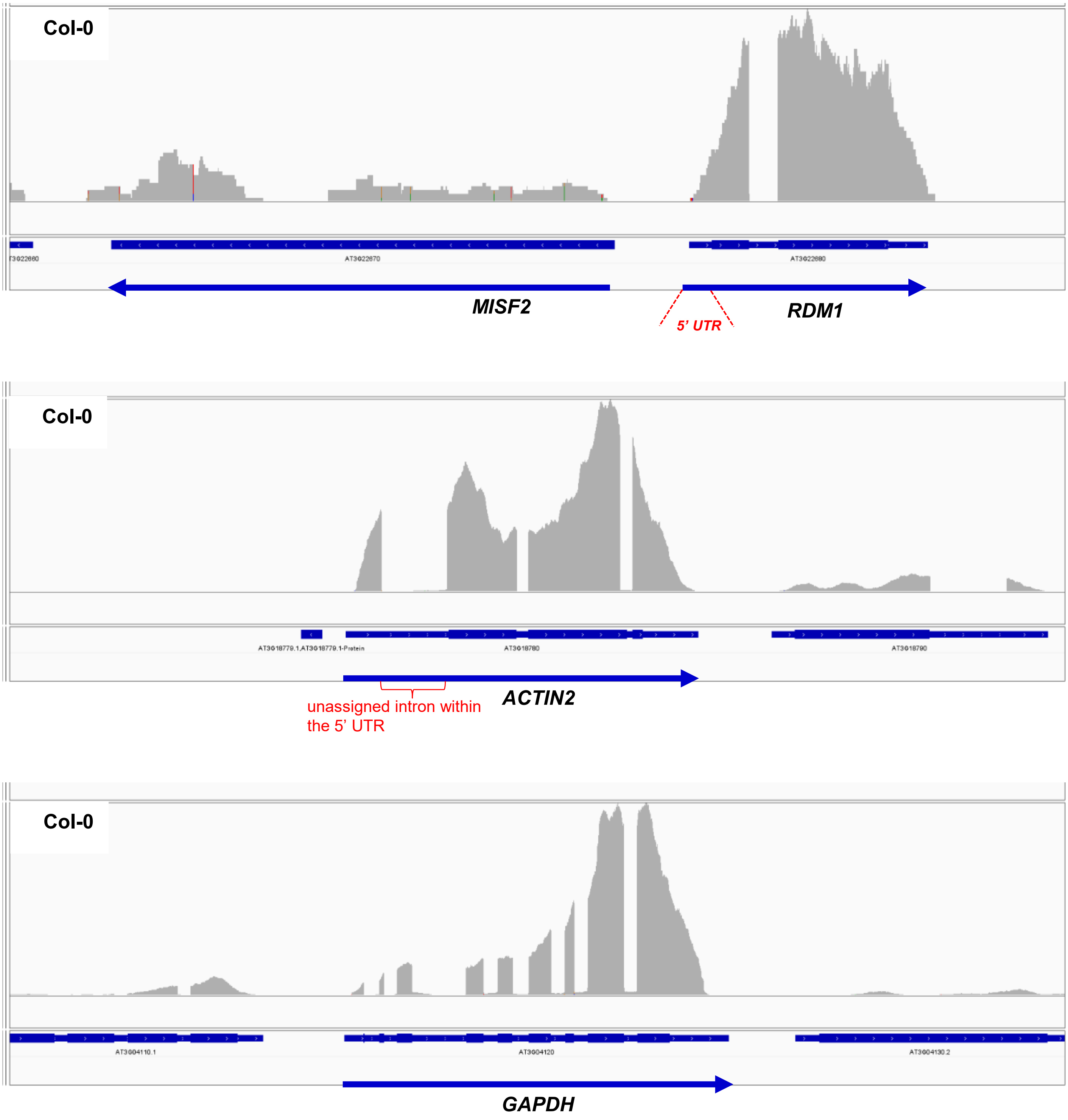

Figure S6. Transcription profiles of *RDM1* gene by RNA-seq analyses. Total RNA extracted from Col-0 plantlets was used to generate Illumina-sequencing (NovaSeq 6000, 150 bp paired-end) libraries (BioProject: PRJNA472433) [84]. Based on these datasets we analyzed the read coverage of *RDM1* (At3g22680) and *MISF2* (At3g22670), as well as *ACTIN2* (At3g18780) and *GAPDH* (At3g04120) genes, in *Arabidopsis thaliana* (Col-0) plants. Thicker boxes indicate to coding sequences (exons), while thinner regions indicate UTR and intron regions in Col-0 plants. The RNA-seq data indicated that RDM1 harbors a 5′UTR of about 50 to 80 nucleotides long. The data also revealed to unannotated introns within the 5′ UTR of *ACTIN2* gene.

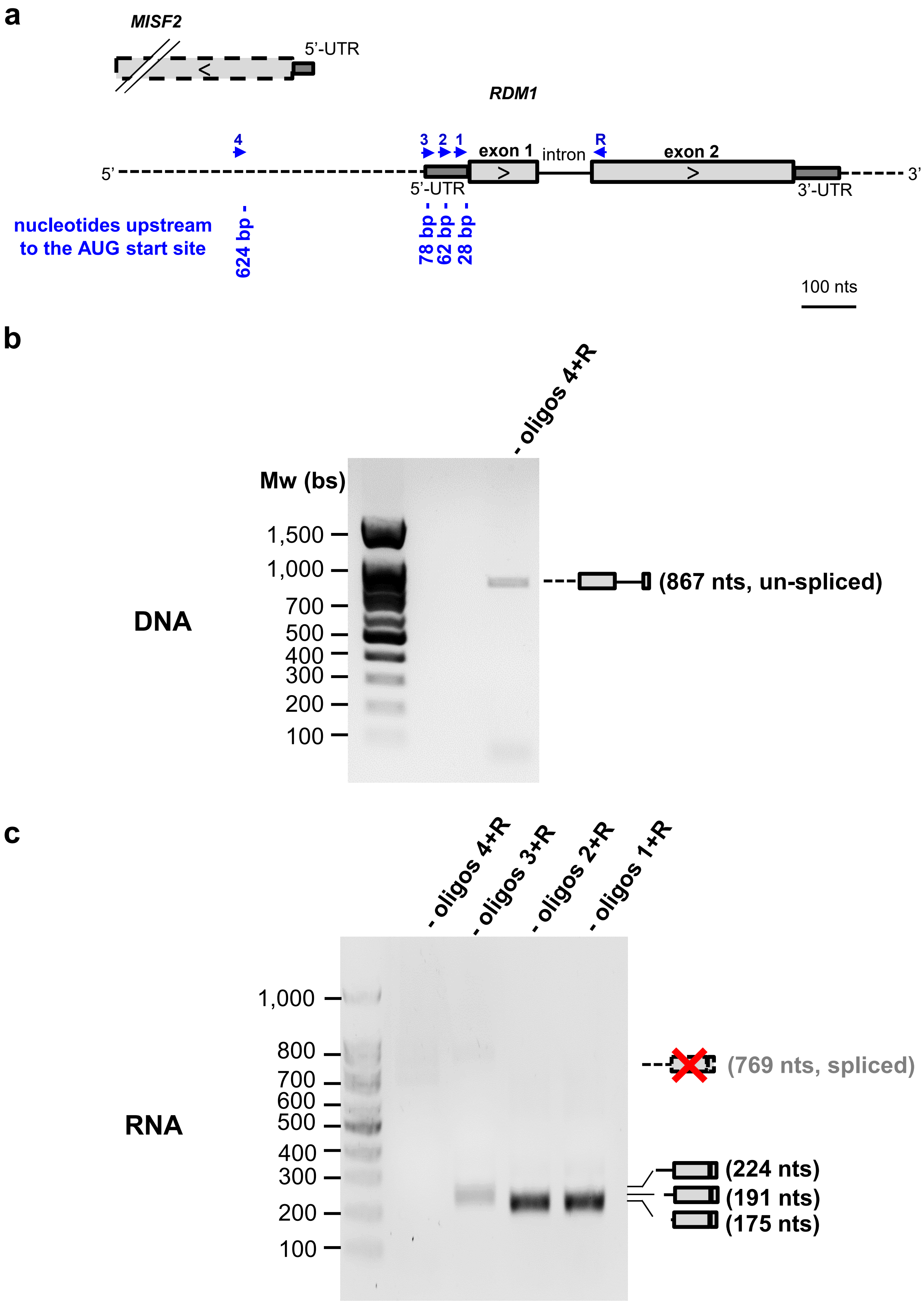

Figure S7. Analysis of the 5′ UTR of *RDM1* transcript by RT-PCR. (a) Scheme of the At3g22670 (*MISF2*) and At3g22680 (*RDM1*) gene-loci and gene structure. The two exons (1 and 2) consist of 124 and 368 nts, respectively, are separated by a 98 bp intron region. The position of the oligonucleotides used in PCR (panel 'b', DNA) and RT-PCR (RNA, panel 'c') are indicated. Oligonucleotides 1, 2 (28 and 62 nts upstream to the initiation codon, respectively) and the reverse primer (R, found at the very 5′ of exon 2) are located inside the coding region of RDM1 gene. Oligonucleotide 3 is found 78 nts upstream to the AUG start codon, while oligonucleotide 4 is found 624 nts upstream to the AUG site (inside the MISF2 coding region).

Table S1. List of oligonucleotides used in this study.

List of oligonucleotides used for the analysis of the mRNA profiles of wild-type and mutant plants by RT-qPCR experiments.

gene target	oligo name	sequence (5'-to-3')
AOX1A	*aox1aF*	AGCATCATGTTCCAACGACGTTTC
	aox1aR	GCTCGACATCCATATCTCCTCTGG
AOX1B	*aox1bF*	GGACCGTGAAATCTCTTCGATGGC
	aox1bR	TCTAGCATCATTGCTCTGCATCCG
AOX1C	*aox1cF*	TCTTCCAGAGGAGGTATGGTTGCC
	aox1cR	AGTGCATAAGCATCCCTCCAACC
AOX1D	*aox1dF*	TTTGCTCGAAGAGGCTGAGAACG
	aox1dR	CTCGTTCGTACCATTTGGGTTGTG
AOX2	*aox2F*	ACGGTGATTCGTGCTGATGAAGC
	aox2R	TCCTTGATTGCGAATGTCAGAAGC
NDA1	*nda1F*	GTATCCAACCGGCGATTTCACG
	nda1R	AGTTACAGTCTCACAATGCACCTC
NDA2	*nda2F*	TGGTGTTGGTCCTTCTCCTTTCG
	nda2R	TCCATTCGTCAATGCCAATCCTTC
NDB1	*ndb1F*	TAACACATTTGGCACTCCTGGTG
	ndb1R	CTCTGTGCATCCTCTACTTCCTTG
atp1	*atp1F*	TCACTTCGACACGTCTTTGC
	atp1R	GGAATGGCCTTGAATCTTGA
atp6	*atp6-1F*	TCTTTTGCGAGTCAATGCAC
	atp6-1R	TCTCGCGTATCTCACATTGC
atp8	*atp8F*	CCGTCGACTTATTGGGAAAA
	atp8R	TTCCTTGGCCATGTACAACA
atp9	*atp9F*	CATTCCCTCTGACGTCGAAT
	atp9R	TCGTCGATTCTTACCCTCGT
atp4	*atp4F*	GGATCAGCTTGCGAATTTGT
	atp4R	GCAAATTGCTTCCCCACTAA
ccmb	*ccmBF*	TCTTGGAATCACATCCAGCA
	ccmBR	CGAGACCGAAATTGGAAAAA
ccmc	*ccmCF*	AGCTACGCGCAAATTCTCAT
	ccmCR	GCCGTGGCGATATAAACAAT
ccmfc	*ccmFcF*	CACATGGAGGAGTGTGCATC
	ccmFcR	GTGGGTCCATGTAAATGATCG
ccmfn-1	*ccmFN1F*	AGCTCTTGGCATTGCTTTGT
	ccmFN1R	AGTGCCACAATCCCATTCAT
ccmfn-2	*ccmFN2F*	CGTGTCGTTCGTAATGGAAA
	ccmFN2R	TGATAAGCCCACCAACTTCC
cob	*cobF*	TGCCGGAATGGTATTTCCTA
	cobR	GCCAAAAGCAACCAAAACAT
cox1	*cox1F*	GTAGCTGCGGTGAAGTAGGC
	cox1R	CTGCCTGGATTCGGTATCAT
cox2	*cox2F*	TGATGCTGTACCTGGTCGTT

	cox2R	TGGGGGATTAATTGATTGGA
cox3	*cox3F*	CCGTAACTTGGGCTCATCAT
	cox3R	AAACCATGAAAGCCTGTTGC
mttb	*mttBF*	GGGGTCTTTCTTTGGAAACC
	mttBR	TCTCCCTCATTCCACTCGTC
nad1 exons a-b	*nad1 1-2F*	GACCAATAGATACTTCATAAGAGACCA
	nad1 1-2R	TTGCCATATCTTCGCTAGGTG
nad1 exons b-c	*nad1 2-3F*	ATTCAGCTTCCGCTTCTGG
	nad1 2-3R	TCTGCAGCTCAAATGGTCTC
nad1 exons c-d	*nad1 3-4F*	AAAAGAGCAGACCCCATTGA
	nad1 3-4R	TCCGTTTGATCTCCCAGAAG
nad1 exons d-e	*nad1 4-5F*	AGCCCGGGATCTTCTTGA
	nad1 4-5R	TCTTCAATGGGGTCTGCTC
nad2 exons a-b	*nad2 exons a-bF*	GCGAGCAGAAGCAAGGTTAT
	nad2 exons a-bR	GGATCCTCCCACACATGTTC
nad2 exons b-c	*nad2 exons b-cF*	AAAGGAACTGCAGTGATCTTGA
	nad2 exons b-cR	AATATTTGATCTTAGGTGCATTTTC
nad2 exons c-d	*nad2 exons c-dF*	GCGCAATAGAAAGGAATGCT
	nad2 exons c-dR	CTATGGGTCTACTGGAGCTACCC
nad2 exons d-e	*nad2 exons d-eF*	CAAAGGAGAGGGGTATAGCAA
	nad2 exons d-eR	TATTTGTTCTTCGCCGCTTT
nad3	*nad3F*	CGAATGTGGTTTCGATCCTT
	nad3R	GCACCCCTTTTCCATTCATA
nad4 exons a-b	*nad4 exons a-bF*	ATTCTATGTTTTTCCCGAAAGC
	nad4 exons a-bR	GAAAAACTGATATGCTGCCTTG
nad4 exons b-c	*nad4 exons b-cF*	AATACCCATGTTTCCCGAAG
	nad4 exons b-cR	TGCTACCTCCAATTCCCTGT
nad4 exons c-d	*nad4 exons c-dF*	TTCCTCCATAAATTCTCCGATT
	nad4 exons c-dR	TGAAATTTGCCATGTTGCAC
nad4L	*nad4L-F*	GGGGAATCCTCCTTAATAGACG
	nad4L-R	AACGAAAATGGCTAACCCAATA
nad5 exons a-b	*nad5 exons a-bF*	TGGACCAAGCTACTTATGGATG
	nad5 exons a-bR	CCATGGATCTCATCGGAAAT
nad5 exons b-c	*nad5 exons b-cF*	TACCTAAACCAATCATCATATC
	nad5 exons b-cR	CTGGCTCTCGGGAGTCTCTT
nad5 exons c-d	*nad5 exons c-dF*	AACTCGGATTCGGCAAGAA
	nad5 exons c-dR	GATATGATGATTGGTTTAGGTA
nad5 exons d-e	*nad5 exons d-eF*	AACATTGCAAAGGCATAATGA
	nad5 exons d-eR	GTTCCTGCGTTTCGGATATG
nad6	*nad6F*	TATGCCGGAAAGGTACGAAG
	nad6R	GTGAGTGGGTCAGTCGTCCT
nad7 exons a-b	*nad7 exons a-bF*	ACCTCAACATCCTGCTGCTC
	nad7 exons a-bR	AAGGTAAAGCTTGAAGATAAGTTTTGT
nad7 exons b-c	*nad7 exons b-cF*	GAGGGACTGAGAAATTAATAGAGTACA
	nad7 exons b-cR	TGGTACCTCGCAATTCAAAA
nad7 exons c-d	*nad7 exons c-dF*	ACTGTCACTGCACAGCAAGC
	nad7 exons c-dR	CATTGCACAATGATCCGAAG

nad7 exons d-e	*nad7 exons d-eF*	GATCAAAGCCGATGATCGTAA
	nad7 exons d-eR	AGGTGCTTCAACTGCGGTAT
nad9	*nad9F*	GGATGACCCTCGAAACCATA
	nad9R	CACGCATTCGTGTACAAACC
rpl2	*rpl2F*	CCGAAGACGGATCAAGGTAA
	rpl2R	CGCAATTCATCACCATTTTG
rpl5	*rpl5F*	AAGGGGTTCGACAGGAAAGT
	rpl5R	CGTATTTCGACCGGAAAATC
rpl16	*rpl16F*	GAGCATTTGCCAAACTCACA
	rpl16R	CGGACACTTTCATCGTGCTA
rps3	*rps3F*	CCGATTTCGGTAAGACTTGG
	rps3R	AGCCGAAGGTGAGTCTCGTA
rps4	*rps4F*	ACCCATCACAGAGATGCACA
	rps4R	TCACACAAACCCTTCGATGA
rps7	*rps7F*	CTCGAACTGAACGCGATGTA
	rps7R	AAGCTGCTTCAAGGATCCAA
rps12	*rps12F*	AGCCAAAGTACGGTTGAGCA
	rps12R	TTTGGGTTTTTCTGCACCAT
matR	*matR-F*	AATTTTTGCGAGAGCTGGAA
	matR-R	TTGAACCCCGTCCTGTAGAC
rrn18	*rrn18F*	CGTCACCTGGGTCAAAAACT
	rrn18R	GCTTGAAAACCGAAGTGAGC
rrn26	*rrn26F*	GACGAGACTTTCGCCTTTTG
	rrn26R	CTTGGAGCGAATTGGATGAT
rrn5	*rrn5F*	CCGACCTCGATATGTGGAATCGTC
	rrn5R	TGGACCATGTCTCCCGAACAATC
18S rRNA (nuclear)	*18S nucl-F*	AAACGGCTACCACATCCAAG
	18S nucl-R	ACTCGAAAGAGCCCGGTATT
ACTIN2 (At3g18780, nuclear)	*actin2-F*	GGTAACATTGTGCTCAGTGGTGG
	actin2-R	AACGACCTTAATCTTCATGCTGC
GAPDH (At3g04120)	*GAPDH-F*	TCTCGATCTCAATTTCGCAAAA
	GAPDH-R	CGAAACCGTTGATTCCGATTC

List of oligonucleotides used for the analysis of the splicing profiles of wild-type and mutant plants by RT-qPCR experiments.

Gene	Forward primer	Reverse primer
rpl2	CCGAAGACGGATCAAGGTAA	CGCAATTCATCACCATTTTG
rpl2 intron exon2	TTAGGAAGAGCCGTACGAGG	CGCAATTCATCACCATTTTG
rps3	AGCCGAAGGTGAGTCTCGTA	CCGATTTCGGTAAGACTTGG
rps3 intron1 exon2	AGCCGAAGGTGAGTCTCGTA	TCTACGGCGGGGTCACTAT
cox2	TGGGGGATTAATTGATTGGA	TGATGCTGTACCTGGTCGTT
cox2 intron1 exon2	TGGGGGATTAATTGATTGGA	AGCAGTACGAGCTGAAAGGC
ccmFc	GTGGGTCCATGTAAATGATCG	CACATGGAGGAGTGTGCATC
ccmFc intron1 exon1	CCCGGATCGAATCAGAGTT	CACATGGAGGAGTGTGCATC
nad1 exon1-2	GACCAATAGATACTTCATAAGAGACCA	TTGCCATATCTTCGCTAGGTG
nad1 intron1 exon2	GACCAATAGATACTTCATAAGAGACCA	CGTGCTCGTACGGTTCATAG
nad1 exon2-3	ATTCAGCTTCCGCTTCTGG	TCTGCAGCTCAAATGGTCTC

nad1 intron2 exon2	GGTTGGGTTAGGGGAACATC	TCTGCAGCTCAAATGGTCTC
nad1 exon3-4	AAAAGAGCAGACCCCATTGA	TCCGTTTGATCTCCCAGAAG
nad1 intron3 exon4	AAAAGAGCAGACCCCATTGA	GGGAGCTGTATGAGCGGTAA
nad1 exon4-5	AGCCCGGGATCTTCTTGA	TCTTCAATGGGGTCTGCTC
nad1 intron4 exon5	AGCCCGGGATCTTCTTGA	ACGGAGCTGCATCCCTACT
nad2 exon1-2	GCGAGCAGAAGCAAGGTTAT	GGATCCTCCCACACATGTTC
nad2 intron1 exon2	GCGAGCAGAAGCAAGGTTAT	CCCATTCCTAACCAGTGGAG
nad2 exon2-3	AAAGGAACTGCAGTGATCTTGA	AATATTTGATCTTAGGTGCATTTTC
nad2 intron2 exon2	CCCGATCCGATAGTTTACAA	AATATTTGATCTTAGGTGCATTTTC
nad2 exon3-4	GCGCAATAGAAAGGAATGCT	CTATGGGTCTACTGGAGCTACCC
nad2 intron3 exon4	GCGCAATAGAAAGGAATGCT	GGCGAATTTCAAACTTGTGG
nad2 exon4-5	CAAAGGAGAGGGGTATAGCAA	TATTTGTTCTTCGCCGCTTT
nad2 intron4exon4	CTTATTCGTGGCAACCTTCC	TATTTGTTCTTCGCCGCTTT
nad4 exon1-2	ATTCTATGTTTTTCCCGAAAGC	GAAAAACTGATATGCTGCCTTG
nad4 intron1 exon2	CCGTATGATGCGGAAGTCTC	GAAAAACTGATATGCTGCCTTG
nad4 exon2-3	AATACCCATGTTTCCCGAAG	TGCTACCTCCAATTCCCTGT
nad4 intron2 exon3	GCGGAACGACCAGAAAAATA	TGCTACCTCCAATTCCCTGT
nad4 exon3-4	TTCCTCCATAAATTCTCCGATT	TGAAATTTGCCATGTTGCAC
nad4 intron3 exon4	TCTAGCTTGGTTCGGAGAGC	TGAAATTTGCCATGTTGCAC
nad5 exon1-2	TGGACCAAGCTACTTATGGATG	CCATGGATCTCATCGGAAAT
nad5 intron1 exon2	TGGACCAAGCTACTTATGGATG	TTCGCAAATAGGTCCGACT
nad5 exon2-3	TACCTAAACCAATCATCATATC	CTGGCTCTCGGGAGTCTCTT
nad5 intron2-exon2	GTACGATCGTGTCGGGTGA	CTGGCTCTCGGGAGTCTCTT
nad5 exon3-4	AACTCGGATTCGGCAAGAA	GATATGATGATTGGTTTAGGTA
nad5 intron3-exon4	AACTCGGATTCGGCAAGAA	GCCGTGTAATAGGCGACCA
nad5 exon4-5	AACATTGCAAAGGCATAATGA	GTTCCTGCGTTTCGGATATG
nad5 intron4 exon5	AACATTGCAAAGGCATAATGA	CCTGTAAACCCCCATGATGT
nad7 exon1-2	ACCTCAACATCCTGCTGCTC	AAGGTAAAGCTTGAAGATAAGTTTTGT
nad7 intron1 exon2	ACGGTTTTTAGGGGGATCTG	AAGGTAAAGCTTGAAGATAAGTTTTGT
nad7 exon2-3	GAGGGACTGAGAAATTAATAGAGTACA	TGGTACCTCGCAATTCAAAA
nad7 intron2 exon3	AGTGGGAGAGCCGTGTTATG	TGGTACCTCGCAATTCAAAA
nad7 exon3-4	ACTGTCACTGCACAGCAAGC	CATTGCACAATGATCCGAAG
nad7 intron3 exon4	TAAAGTGAAGTGGTGGGCCT	CATTGCACAATGATCCGAAG
nad7 exon4-5	GATCAAAGCCGATGATCGTAA	AGGTGCTTCAACTGCGGTAT
nad7 intron4 exon5	CGGCCAAATGACTACAGGAT	AGGTGCTTCAACTGCGGTAT

List of other oligonucleotides used for the analysis of *MISF2* and *RDM1* genes, the construction of MISF2-GFP and RACE analyses.

**Gene**	**Forward primer**	**Reverse primer**
MISF2 (AT3G22670)	ATGCTCACTAAGCTTAGGATTA	ATTATAAGTATGACCTGAATGCA
MISF2.1 (SALK_067654)	ATTTTGCCGATTTCGGAAC	ATTATAAGTATGACCTGAATGCA
MISF2.2 (SALK_066141)	ATTTTGCCGATTTCGGAAC	ATTATAAGTATGACCTGAATGCA
MISF2-GFP (*misf2*-B1+ *misf2*-B2)	GGGGACAAGTTTGTACAAAAAAGC AGGCTAAAATGCTCACTAAGCTTAG GATTAGT	GGGGACCACTTTGTACAAGAAAG CTGGGTCAAGCGTAACAAGCTTA GATTCTTC
MISF2-RACE_S1	AACAATCGGCGACTCCTCTG	AGGATGGAGGATGAGGAGGG
MISF2-promo-B1 (F) *MISF2-Cpl-B2 (R)*	GGGGACAAGTTTGTACAAAAAAGCAGGCT AAAGCTTCAATGCTTGAACAAGGTTTC	GGGGACCACTTTGTACAAGAAAG CTGGGTCCGAAACAGATAAAGGA CTGTGAG
MISF2-B1 (F) *MISF2-Cpl-B2 (R)*	GGGGACAAGTTTGTACAAAAAAGCAGGCT AAAATGCTCACTAAGCTTAGGATTAGT	GGGGACCACTTTGTACAAGAAAG CTGGGTCCGAAACAGATAAAGGA CTGTGAG
RDM1-GSP	GATTACGCCAAGCTTCAGCGATGGC TTTTCACCGCTCGAT	GATTACGCCAAGCTTTCAACGAG CCACGATTCGTCGGGAT
RDM1-NGSP	GATTACGCCAAGCTTCTTCTCACAG AGACGTCGCTGATGAAG	GATTACGCCAAGCTTGCTTAATCC AACCCAGCTTGTGAACGGA
RDM1-RT-PCR1	GTCAAAATCTAACTGCTCGACAG	CTCTGCTCTTCTCAGCAACGAAC
RDM1-RT-PCR2	GTCATCGTCTTCGTACCTTTCTGC	CTCTGCTCTTCTCAGCAACGAAC
RDM1-RT-PCR3	AAGGAACCTCGTGTGGTCATC	CTCTGCTCTTCTCAGCAACGAAC
RDM1-RT-PCR4	CCACTGGTTTCTTCACTACATTTCCC	CTCTGCTCTTCTCAGCAACGAAC

Table S2. List of antibodies used in this study.

Antibody	Protein I.D.	origin	serum	dilution	Reference / source
AtpA	Mitochondrial ATP-synthase subunit α	*Zea mays*	Mouse (monoclonal)	1/500	Thomas Elthon collection, PM014
AtpB	Mitochondrial ATP-synthase subunit β	*Zea mays*	Mouse (monoclonal)	1/5,000	(Michael *et al.* 1993)
CA2	γ-carbonic anhydrase-like subunit 2	*Arabidopsis thaliana*	Rabbit (polyclonal)	1/1,000	(Perales *et al.* 2005, Sunderhaus *et al.* 2006)
Cox2	Cytochrome oxidase subunit-2	*Arabidopsis thaliana*	Rabbit (polyclonal)	1/5,000	Agrisera antibodies, AS04 053A
Nad1	NADH-dehydrogenase complex subunit-1	*Arabidopsis thaliana*	Rabbit (polyclonal)	1/1000	Gift of Dr. Etienne Meyer, Halle U.
Nad9	NADH-dehydrogenase complex subunit-9	*Triticum spp.*	Rabbit (polyclonal)	1/50,000	(Lamattina *et al.* 1993)
RISP	Rieske iron-sulfur protein	*Arabidopsis thaliana*	Rabbit (polyclonal)	1/5,000	Gift of Prof. Ian Small, UWA
VDAC (PORIN)	Mitochondrial membrane-associated β–barrel proteins	*Zea mays*	Mouse (monoclonal)	1/1000	Thomas Elthon collection, PM035
AOX1/2	Alternative oxidase	*Sauromatum guttatum*	Rabbit (polyclonal)	1/500	Agrisera antibodies, AS04 054
Nad6	NADH-ubiquinone oxidoreductase chain 6	*Arabidopsis thaliana*	Rabbit (polyclonal)	1:1000	Agrisera (AS15 2926)
Cob	Apocytochrome B	*Beta vulgaris* ssp. *maritima*	Rabbit (polyclonal)	1:4000	Meyer et al. 2018
CYTC1	Cytochrome C1	*Arabidopsis thaliana*	Rabbit (polyclonal)	1:1000	Giégé et al. 2005
CYTC	Cytochrome C	*Arabidopsis thaliana*	Rabbit (polyclonal)	1:1000	Agrisera (AS08 343A)

References

Giege. P, Sweetlove LJ, Cognat V, Leaver CJ. 2005. Coordination of nuclear and mitochondrial genome expression during mitochondrial biogenesis in Arabidopsis. Plant cell, **17**, 1497-1512.

Lamattina, L., Gonzalez, D., Gualberto, J. and Grienenberger, J.-M. (1993) Higher plant mitochondria encode an homologue of the nuclear-encoded 30-kDa subunit of bovine mitochondrial complex I. *Eur J Biochem*, **217**, 831-838.

Meyer EH, Lehmann C, Boivin S, Brings L, De Cauwer I, Bock R, Kuhn K, Touzet P. (2018) CMS-G from Beta vulgaris ssp. maritima is maintained in natural populations despite containing an atypical cytochrome c oxidase. *Biochem J,* **475**, 759–773.

Michael, H.L., Arnost, H. and Elthon, T.E. (1993) Monoclonal Antibodies to the a- and b- Subunits of the Plant Mitochondrial F_1-ATPase. *Plant Physiology*, **101**, 931-937.

Perales, M., Eubel, H., Heinemeyer, J., Colaneri, A., Zabaleta, E. and Braun, H.-P. (2005) Disruption of a nuclear gene encoding a mitochondrial gamma carbonic anhydrase reduces complex I and supercomplex I+III2 levels and alters mitochondrial physiology in Arabidopsis. *J Mol Biol*, **350**, 263-277.

Sunderhaus, S., Dudkina, N.V., Jansch, L., Klodmann, J., Heinemeyer, J., Perales, M., Zabaleta, E., Boekema, E.J. and Braun, H.P. (2006) Carbonic anhydrase subunits form a matrix-exposed domain attached to the membrane arm of mitochondrial complex I in plants. *J Biol Chem*, **281**, 6482-6488.
